# Design of nano *mume Fructus* charcoal by reshaping inflammatory microenvironment as biofunctional wound nanomedicine

**DOI:** 10.1016/j.mtbio.2025.101971

**Published:** 2025-06-09

**Authors:** Pan Liang, Yining Ma, Menglin Song, Hong Wang, Xin Peng, Qin Sun, Hongping Shen, Zengjin Liu, Pei Luo, Wei Ren

**Affiliations:** a Drug Research Center of Integrated Traditional Chinese and Western Medicine, The Affiliated Traditional Chinese Medicine Hospital, Southwest Medical University, Luzhou, 646000, China; b State Key Laboratories for Quality Research in Chinese Medicines, Faculty of Chinese Medicine, Macau University of Science and Technology, Macau 853, China

**Keywords:** *Mume Fructus* charcoal, Biofunctional nanomedicine, Wound repair, Hemostasis, Inflammatory microenvironment

## Abstract

Hemorrhagic wound management in severe trauma patients remains a vital clinical problem because of uncontrolled bleeding and inflammatory responses. Charcoal drugs have shown great potential for facilitating hemostasis and wound repair for more than two thousand years. Nevertheless, studies on the inherent biological activities of charcoal drugs and their corresponding pharmacological effects are far from satisfactory. Herein, a novel carbon dot prepared from charred *Mume Fructus* (CMF-CDs) for enhancing hemorrhagic wound healing was reported for the first time. Surprisingly, the as-prepared CMF-CDs showed good biocompatibility, reactive oxygen species scavenging ability and protection of human cells from oxidative damage. Inspired by this, we confirmed that CMF-CDs enhanced hemorrhagic wound healing by facilitating hemostasis, M2-type macrophage polarization, angiogenesis, collagen deposition and tissue regeneration. Proteomic results further revealed that the CMF-CDs reshaped the inflammatory microenvironment of the wound by reducing excessive ROS produced by tissue oxidative phosphorylation and the tricarboxylic acid cycle metabolic pathway. Ultimately, the discovery of CMF-CDs holds enormous promise for managing severe traumatic wounds and provides theoretical and material insights into charcoal drug-mediated hemostasis applications.

## Graphical abstract

**Figure:**
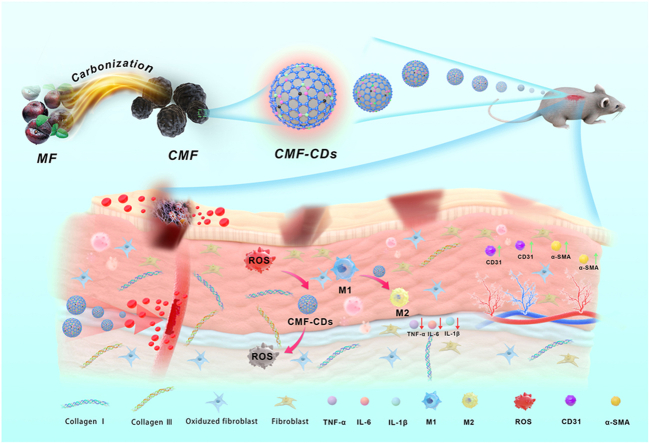

## 1 Introduction

Hemorrhagic wounds from traffic accidents, natural disasters, free weapons and battlefield explosions are serious problems for civilian and military trauma patients worldwide and impose a considerable economic and social burden [1,2]. Wound healing is a sequential and continuous process that dynamically involves hemostasis, inflammation, proliferation and remodeling of four overlapping cascades [3]. Rapid and effective hemostasis by reducing blood loss and shortening bleeding time is the first and most important step during hemorrhagic wound healing [4]. However, conventional hemostatic materials struggle to penetrate into tissue and often cause an uncontrolled immune response, leading to unsatisfactory outcomes [5]. In addition, large hemorrhagic wounds are prone to severe inflammation, manifested by the uncontrolled accumulation of reactive oxygen species (ROS) and proinflammatory cytokines and hindered angiogenesis and granulation tissue formation [6]. Excess ROS, such as hydroxyl radicals (·OH), superoxide anions (·O_2_^−^) and hydrogen peroxide (H_2_O_2_), can damage important cellular components and increase inflammation, eventually impeding wound healing [7]. In response to the inflammatory microenvironment, macrophages differentiate into proinflammatory macrophages (M1 type) and secrete many proinflammatory cytokines in the early stage of inflammation [8]. In contrast, M2 macrophages help suppress inflammation and promote skin regeneration and wound repair. Due to the increased inflammation in hemorrhagic wounds, macrophages tend to polarize into the M1 phenotype and continuously recruit and activate proinflammatory cells; thus, they fail to heal [9]. Hence, a promising wound dressing should synergistically combine multiple functionalities, including hemostasis, elimination of excess ROS and regulation of the inflammatory microenvironment, thereby effectively accelerating wound repair.

To date, an increasing attention has been given to nanozymes, which are nanomaterials with enzyme-like characteristics that have been employed as substitutes for natural enzymes in various applications [10]. Nanozymes have been applied in wound treatment because of their ability to regulate the ROS-dependent inflammatory microenvironment. For example, Liu et al. developed ultrasmall Cu5.4O nanoparticles (Cu5.4O USNPs), and found that Cu5.4O USNPs possessed multiple mimetic enzyme properties to exhibit cytoprotective effects against ROS-mediated organ injury at very low doses [11]. Ge et al. constructed a multilayered protective nanoarmor (NA) for IL-4 delivery (IL-4@PEGRA NAs), which showed their strong anti-inflammatory effects by scavenge ROS with the specific release of IL-4, promoting M2-polarization of macrophage and downregulating the associated inflammatory mediators [12]. Moreover, Shi et al. reported self-assembled micelles of P311 peptides and deblock copolymer and discovered that they have unique ability to promote the formation of proregenerative microenvironment in wound tissue while also providing cues for epidermal cell migration [13]. However, despite the effectiveness of nanozyme-based therapies, several limitations of the reported nanozymes still impede their clinical application, including complex preparation, high cost and poor biocompatibility. In particular, some metal-based nanozymes, including Au, Mn, Pd, Cd, Pt, and Ag, exhibit potential health hazards [14]. As a novel type of nanozyme, carbon dots (CDs) have shown a great potential as an antioxidant to remove excess ROS on wound surfaces. Ultrasmall endow CDs have abundant active sites and multiple functional groups [10]. Possibly owing to adduct formation, electron transfer, and hydrogen supply, CDs show intrinsic antioxidant activities and the ability to scavenge ROS [1]. CDs synthesized from natural Chinese medicine are considered to be highly biocompatible and negligible toxicity and have been used for in vivo therapeutic applications. For example, Deng et al. reported honeysuckle-derived CDs (Hy-CDs) with SOD-like enzymatic activities can effectively reduce oxidative stress and inhibit the secretion of pro-inflammatory cytokines [15]. CDs derived *Ligusticum wallichii* [16], Camelina [1], and *Cannabis sativa* L. seeds [17] have been successfully developed for wound repair. As mentioned above, the current researches associated with CDs are characterized by superior biocompatibility and high antioxidant capacity, it remains a challenge to develop multifunctional CDs for hemorrhagic wounds from natural biomass materials.

Charcoal drugs are a class of highly distinctive natural products, and their hemostatic efficacy was confirmed in Chinese medicine clinics as early as two thousand years [18]. The application of charcoal drugs to treat diseases is an interesting option and has shown a growing trend, especially in recent years [19]. *Mume Fructus* (MF, Wumei in Chinese) is processed by drying and blackening the fruit of *Prunus mume* Sieb. et Zucc, a medicinal plant in the Rosaceae family [20]. According to the 2020 Chinese Pharmacopoeia, MF is a well-known medicinal food homologous to natural products that is usually used to treat inflammation-related diseases such as diarrhea, chronic cough and colitis [21]. Charred *Mume Fructus* (CMF, Wumeitan in Chinese) is the charred product of MF and has been widely applied in clinical practice for blood in the stool, metrorrhagia and hypospadias, chronic diarrhea and chronic dysentery [22]. Notably, raw MF has no hemostatic effect but shows increased hemostasis after charring [23]. Inspired by the similar high-temperature processing between charcoal drug carbonization and CD pyrolysis, we successfully prepared CDs from CMF and named them CMF-CDs. Unexpectedly, ultrasmall CMF-CDs are rich in abundant hydroxyl, carbonyl and carboxyl groups, contributing to their significant catalytic performance of superior superoxide dismutase (SOD), catalase (CAT) and glutathione peroxidase (GPx) as well as their strong antioxidant ability. Therefore, the CMF-CDs exhibited high biocompatibility and improved cell healing. Remarkably, we demonstrated the fast and effective hemostatic efficacy of CMF-CDs in rat liver and tail injury models. *In vitro*, CMF-CDs improved the survival of RAW 264.7 cells under oxidative stress, as indicated by their obvious ROS-scavenging activity. By employing hemorrhagic wounds as an in vivo model, we further found that CMF-CDs not only promoted M2 macrophage polarization and decreased the levels of proinflammatory factors (TNF-α, IL-1β, and IL-6) but also enhanced angiogenesis, collagen deposition and follicle regeneration, thus facilitating wound repair. Proteomic analysis further revealed that CMF-CDs effectively alleviated the inflammatory environment and facilitated wound healing by interfering with oxidative phosphorylation and tricarboxylic acid cycle metabolic pathways. Overall, we developed a natural CMF-CDs as an accelerant for hemorrhagic wounds, highlighting the promising value of charcoal-based drugs in modern medicine.

## 2 Results and discussion

### 2.1 Synthesis and characterization of CMF-CDs

**Fig. 1:**
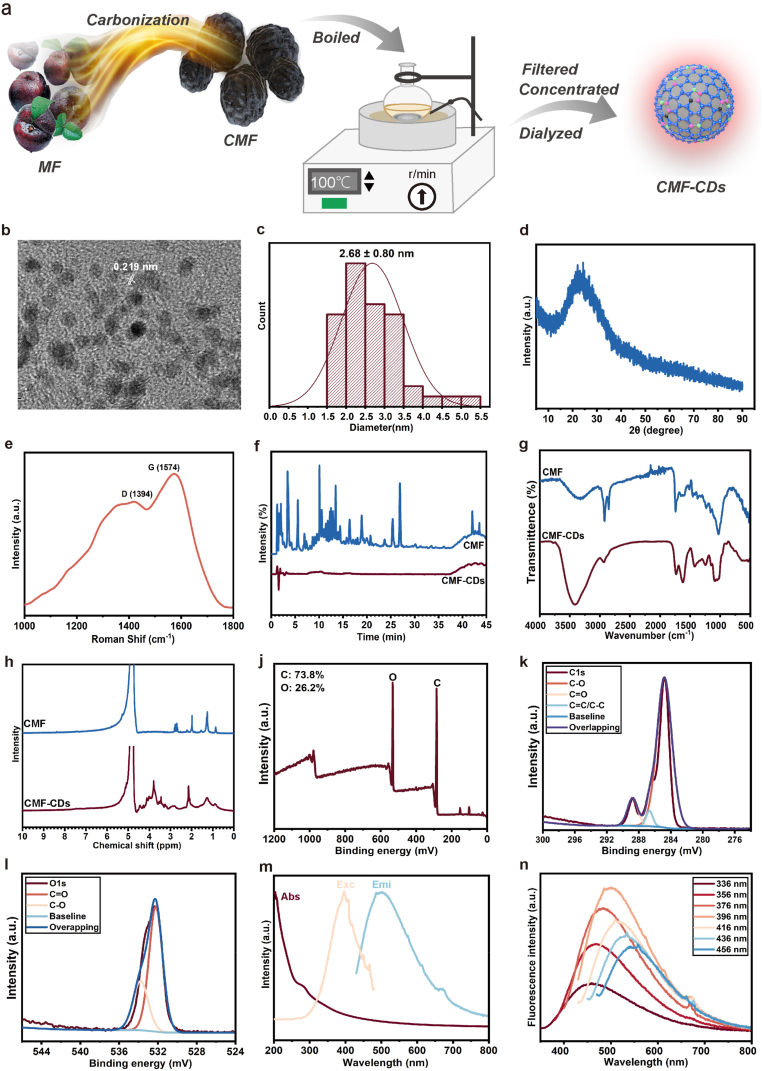
Synthesis and characterization of CMF-CDs. a) Schematic illustration of CMF-CD synthesis. b) High-resolution TEM image, c) size distribution, d) XRD pattern, e) Raman spectrum, h) ^1^H nuclear magnetic resonance spectrum, l) UV–vis absorption (Abs), fluorescence emission (Exc) and excitation (Emi) spectra, and m) fluorescence emission spectra under 336–456 nm excitation of the prepared CMF-CDs. f) UHPLC-HR-MS and g) FT-IR spectra of CMF and CMF-CDs. i) XPS survey, high-resolution XPS of j) C1s, k) O1s with identification of peaks by curve fitting.

Inspired by charcoal drug processing, CMF-CDs with high water solubility were easily prepared by heating CMF in the aqueous phase (Fig. 1a). After separation and purification, the obtained CMF-CDs exhibited a spherical shape with a uniform size of 2–5 nm according to high-resolution transmission electron microscopy (HR-TEM) images (Fig. 1b and c). Interestingly, the CMF-CDs had a lamellar structure with a lattice spacing of 0.219 nm, corresponding to the (100) lattice plane of graphite [24]. The diffraction peak of the CMF-CDs that appeared in the X-ray diffraction (XRD) pattern was at approximately 24.86°, which is consistent with the (002) facet of graphite (Fig. 1d) [25]. Therefore, we concluded that the CMF-CDs were consisted of sp^2^ carbon clusters [26]. In the Raman spectrum of the CMF-CDs, the D-band and G-band of graphene were two peaks at 1394 and 1574 cm^−1^, respectively (Fig. 1e). The intensity ratio between the D-band and G-band was 0.79, implying that a large number of defects existed on the surface of the CMF-CDs [25]. According to the ultrahigh-performance liquid chromatography-high-resolution mass spectrometry (UHPLC-HR-MS) results, CMF are rich in complex chemical components (Fig. 1f). In contrast, the CMF-CDs had no small molecular compounds under the same conditions, suggesting the high purity and homogeneity of the CMF-CDs. Thus, the above characterization indicated that CMF-CDs containing sp^2^ networks were successfully synthesized [27].

To gain insight into the chemical structure of the CMF-CDs, Fourier transform infrared (FT-IR) spectroscopy was further used to identify chemical bonds. As presented in Fig. 1g, in the FT-IR spectra of the CMF-CDs, five typical absorption peaks appeared at 3416, 2932, 1620, 1415 and 1083 cm^−1^, corresponding to -O-H, -C-H, -C=C-, -C=O and -C-O-, respectively, suggesting that the CMF-CDs were rich in hydroxyl, carboxyl and carbonyl groups on their surfaces. The FT-IR spectra showed that the CMF-CDs contained some functional groups, which is consistent with our previous study [5]. According to the ^1^H nuclear magnetic resonance (^1^H NMR) spectra of the CMF-CDs, the chemical shift between 1.0 and 1.3 ppm indicated the presence of aliphatic hydrocarbons. The peaks ranging from 2.2 to 2.8 ppm were attributed to the α-H of the carboxyl group, while the peaks of ^1^H in the hydroxyl and carbonyl groups were observed in the range of 3.3–4.5 ppm (Fig. 1h). Furthermore, X-ray photoelectron spectroscopy (XPS) analysis indicated that the CMF-CDs consisted of 73.8 % C and 26.2 % O (Fig. 1i). The binding energy peaks at 288.77, 286.67 and 284.80 eV in the high-resolution C1s spectrum corresponded to -C=O, -C-O- and -C=C−/−C-C- groups, respectively, further supporting that the CMF-CDs possessed -C=O and -C-O-C- groups (Fig. 1j). The high-resolution O1s spectrum was fitted with two characteristic peaks at 532.21 and 533.78 eV, assigned to the -C-O- and -C=O- groups, respectively (Fig. 1k). Subsequently, the optical properties of the CMF-CDs were evaluated. As presented in Fig. 1l, the π-π∗ electron transition was reflected in the characteristic absorption peak at 283 nm in the UV–vis spectrum. In the fluorescence spectrum, the CMF-CDs showed a maximum emission peak at 502 nm under the maximal excitation wavelength of 396 nm. Moreover, the excitation-dependent emission of the CMF-CDs increased as the excitation wavelength increased from 336 to 456 nm (Fig. 1m). The UV–vis and fluorescence spectra were consistent with those of previously reported Chinese herb-derived CDs [18,28]. Changes in ζ-potential can reflect the stability of the CDs. The higher ζ-potential contributes to the denser surface charge and greater electrostatic repulsion between particles, thereby enhancing the stability [29]. In this study, pH = 7.4 PBS and pH = 7.4 PBS containing 10 % FBS were used to simulate in vitro and in vivo environments, respectively. As presented in Fig. S1, the ζ-potential changes observed during the 14-day placement period were negligible, with approximately −16 mV, suggesting good stability of CMF-CDs both in vitro and in vivo.

In this work, CMF-CDs of ultrasmall size containing abundant functional groups such as hydroxyl, carboxyl and carbonyl groups on their surface were directly extracted from CMF. The carbonization of charcoal drugs is similar to the pyrolysis of CDs. Pyrolysis is the direct thermal degradation of precursors under anoxic conditions and is very suitable for the preparation of charcoal drugs [19]. In comparation to other methods for preparation, a higher reaction temperature and shorter heating time are generally required, which remarkably affects the structure of CDs [30]. One hypothesis is that the various properties of CDs, such as functional groups, size and solubility, play a vital role in their biological activity [31]. Currently, the pyrolysis temperature and reaction time of many charcoal drugs are approximately 300–350 °C and 1 h, respectively [32]. The particle size of charcoal drug-derived CDs prepared by pyrolysis is approximately 5 nm under the existing synthesis conditions, showing excellent stability, water solubility and physiological barrier penetration [33]. The reaction mechanism of synthetic charcoal drug-derived CDs is a complicated process. Charcoal drugs are rich in various sugars, acids, phenolic compounds and their glycosides, which can be dehydrated at high temperatures to form small furfural molecules and carbonized to form a carbon core [18]. In addition, the active components contained in charcoal drugs participate in the nucleation and surface functional modification of CDs by connecting surface functional groups through covalent or noncovalent bonds [34]. Therefore, from another perspective, there may be CDs in the charcoal drugs themselves, and the conditions for pyrolysis are stricter than those for the ordinary preparation of charcoal drugs. This finding also confirms the rationality of using Chinese medicines as precursors to prepare CDs owing to the long history of charcoal drugs in clinical applications. Nevertheless, the relationships between the inherent bioactivity of charcoal drug-derived CDs and their potential pharmacological effects have received inadequate attention and become a “blind area”.

### 2.2 ROS scavenging activity of the CMF-CDs

**Fig. 2:**
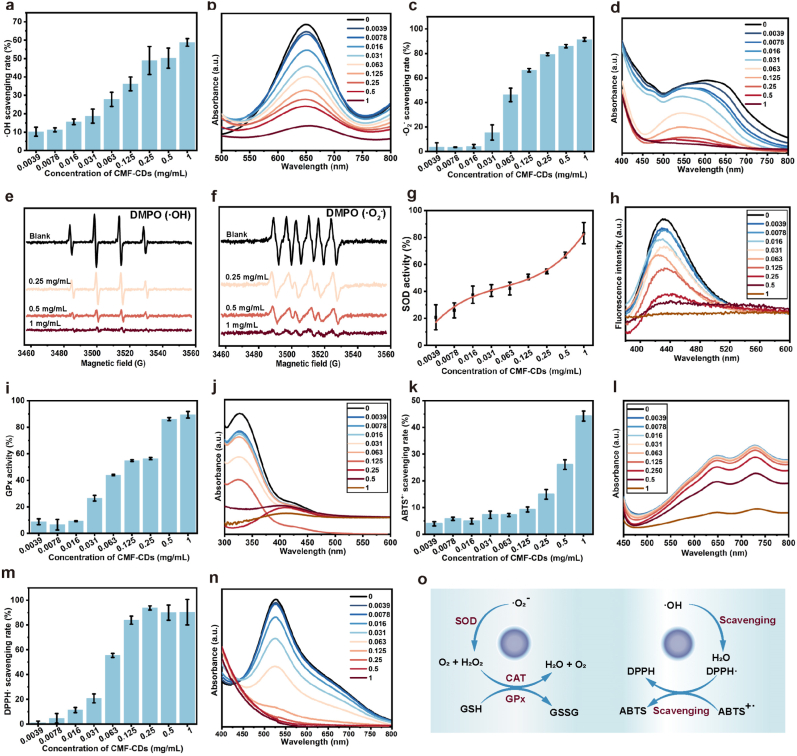
Evaluation of the ROS scavenging activity of CMF-CDs. a) ·OH scavenging rate of CMF-CDs. b) UV–vis spectra of TMB treated with various concentrations of CMF-CDs in the presence of H_2_O_2_ and FeSO_4_. c) ·O_2_^−^ scavenging rate of CMF-CDs. d) UV–vis spectra of NBTs treated with various concentrations of CMF-CDs upon light irradiation. ESR spectra of e) ·OH and f) ·O_2_^−^ trapped by DMPO after CMF-CD treatment. g) SOD-like activity of CMF-CDs. h) Fluorescence spectra of TPA reflecting the CAT-like activity of CMF-CDs. i) GPx-like activity of CMF-CDs. j) UV–vis spectra of GSH and benzoic acid treated with CMF-CDs. k) ABTS^+·^ scavenging rate of CMF-CDs. l) UV–vis spectra of ABTS^+·^ treated with CMF-CDs. m) DPPH· scavenging rate of CMF-CDs. n) UV–vis spectra of DPPH· treated with CMF-CDs. o) Diagram showing the multizyme-like activities of CMF-CDs.

Three representative ROS, ·OH, ·O_2_^−^ and H_2_O_2,_ were examined to investigate the enzymatic catalytic activities of the CMF-CDs. To determine the ·OH scavenging efficiency of the CMF-CDs, a 3,3′,5,5′-tetramethylbenzidine (TMB) chromogenic test was performed. The CMF-CDs inhibited TMB oxidation in a concentration-dependent manner by removing ·OH produced from the Fenton reaction between H_2_O_2_ and FeSO_4_. As a result, approximately 90 % of the total ·OH was decomposed by the 1 mg/mL CMF-CDs (Fig. 2a and b). Next, the ·O_2_^−^ scavenging activity of the CMF-CDs was assessed by the nitro blue tetrazolium (NBT) photoreduction method. Upon light irradiation, NBT was reduced to blue methyl hydrazine by ·O_2_^−^ and exhibited a maximum absorption wavelength of 560 nm [35]. After the addition of CMF-CDs, the photoreduction process of NBT showed concentration-dependent behavior, represented by the reduced absorbance of blue methyl hydrazine (Fig. 2c and d). Moreover, using 5,5-dimethyl-1-pyrroline A′-oxide (DMPO) as the spin trapping reagent, electron spin resonance (ESR) spectroscopy was applied to further confirm the ·OH and ·O_2_^−^ scavenging activities of the CMF-CDs (Fig. 2e and f). Next, the concentration-dependent SOD-like activity of CMF-CDs was determined using the 2-(4-iodophenyl)-3-(4-nitrophenyl)-S-(24disulfophenyl)-2H-tetrazolium (WST-1) method. Surprisingly, the inhibitory effect of CMF-CDs on formazan generation was close to 95 % when the concentration reached 1 mg/mL, indicating that the CMF-CDs had favorable SOD-mimicking enzyme activity (Fig. 2g). This activity could be due to the abundance of hydroxyl, carboxyl and carbonyl groups containing on the CMF-CDs surface, which are conducive to the formation of hydrogen bonds as the active sites for trapping ·O_2_^−^ [36]. Moreover, H_2_O_2_, which has a long half-life and strong cell membrane permeability, displayed the greatest significance among the three representative ROS [11]. Therefore, we further focused on the catalase (CAT)-like and glutathione peroxidase (GPx)-like activities of CMF-CDs, which are responsible for H_2_O_2_ elimination. The CAT-like activity of the CMF-CDs was evaluated using terephthalic acid (TPA), which can react with H_2_O_2_ to generate 2-hydroxyterephthalic acid with high fluorescence. As shown in Fig. 2h, the fluorescence intensity reduced gradually with increasing CMF-CDs concentration, indicating CAT-like activity. In addition, CMF-CDs also displayed concentration-dependent GPx-like activity, scavenging H_2_O_2_ and catalyzing the oxidation of reduced glutathione (Fig. 2i and j) [37]. To further confirm the antioxidant activity of the CMF-CDs, a 2,2′-azino-bis(3-ethylbenzothiazoline 6-sulfonate) (ABTS) test was performed. As shown in Fig. 2k, approximately half of the ABTS^+·^ was eliminated by the 1 mg/mL CMF-CDs. In addition, the UV–vis absorbance of ABTS^+·^ decreased with increasing CMF-CD concentration (Fig. 2i). In addition to eliminating ROS, 2,2-diphenyl-1-(2,4,6-trinitrophenyl) hydrazyl (DPPH) was selected to investigate the removal of reactive nitrogen species (RNS). Similarly, the CMF-CDs sufficiently scavenged DPPH in a concentration-dependent manner (Fig. 2m and n). Overall, we concluded that the ROS and RNS elimination ability of the CMF-CDs may be attributed to their intrinsic multienzyme-like properties (Fig. 2o).

As previously reported, CDs with SOD-like activity enable them to effectively eliminate ROS and thus reduce oxidative damage, which is attributed to their potential in therapeutic interventions for inflammation-related diseases [38]. Due to the unique structural properties of CDs, such as abundant oxygen and nitrogen functional groups coupled with electronic conditions, the interaction of CDs with enzymes can change the enzyme structure and catalyze the process [11]. In addition, ultrasmall CDs provide numerous binding and catalytic sites for catalytic reactions. They also have the advantages of natural enzyme-like activity, easy preparation, strong stability, good biocompatibility and cell permeability [39]. The present work further confirmed the remarkable multienzyme-like properties of CMF-CDs, promoting the effective scavenging of oxidative free radicals. Research on how CDs affect the catalytic activity of enzymes remains unsatisfactory. By simulating the active sites catalyzed by various enzymes, diverse functional groups on the surface of CDs have been shown to offer excellent biological capabilities [40]. Increasing evidence suggests that CD-based nanozymes, especially SOD, CAT and GPx nanozymes, simulate the structure and function of natural enzymes, contributing to eliminate ROS and prevent oxidative damage [27]. For example, through surface modification, it was confirmed that the catalytic mechanism of CDs-based SOD-like activity was associated with the binding of surface functional groups with ·O_2_^−^, which facilitates electron transfer between CDs and ROS and ultimately accelerates destruction of free radicals [41]. This CD nanozyme could efficiently penetrate cells and accumulate in mitochondria by scavenging ROS and reducing proinflammatory factors [25]. In this study, CD nanozymes were prepared from CMF, a distinctive Chinese medicine that is undergone high-temperature carbonization. As reported, charcoal drug-derived CDs have a large specific surface area and sp^2^ nucleation properties and could be bound to a variety of molecules by π–π∗ superposition and electrostatic interactions [42]. Therefore, it can be inferred that charcoal drugs hold great potential as excellent precursors for nanozymes.

### 2.3 Biocompatibility, cell scratch healing and hemostatic properties of the CMF-CDs

**Fig. 3:**
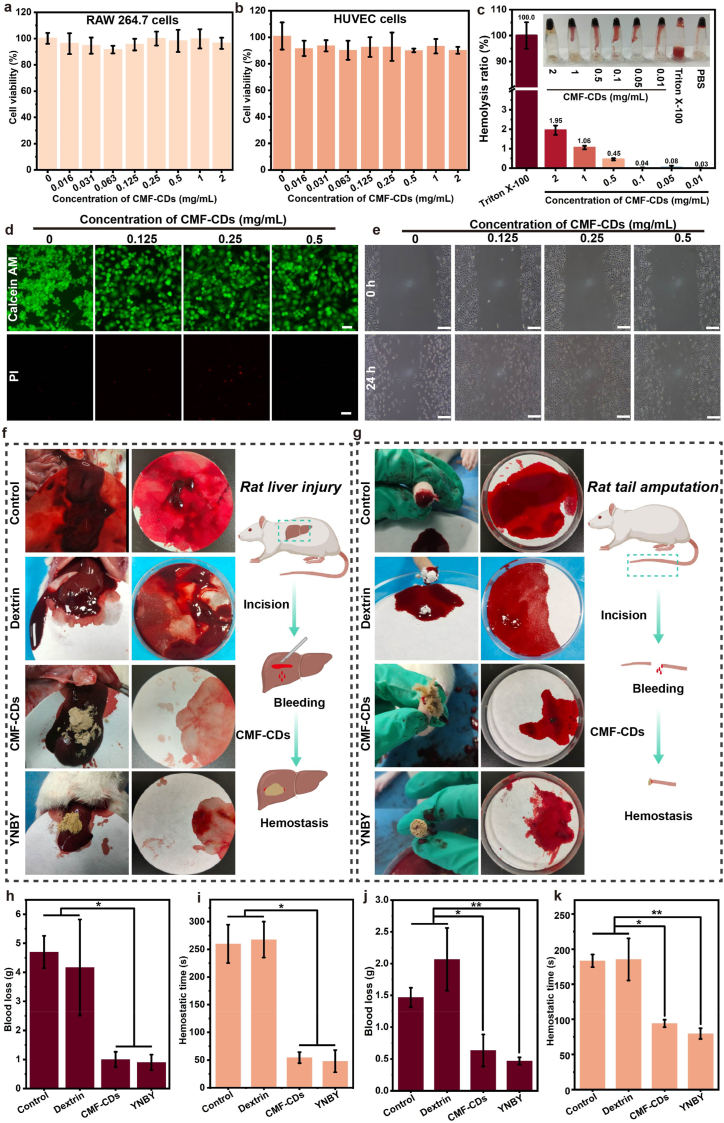
Biocompatibility, cell scratch healing and hemostatic properties of the CMF-CDs. Viability of a) RAW 264.7 cells and b) HUVECs treated with different concentrations of CMF-CDs (n = 6). c) Hemolysis rate of red cells (n = 3, insert representative image). d) Live/dead cell staining after incubation with different concentrations of CMF-CDs for 24 h (scale bar: 100 μm, n = 3). e) Images of RAW264.7 migration by a scratch wound assay after 24 h of incubation with CMF-CDs (scale bar: 200 μm). Illustration and diagrams of hemostasis evaluation in the f) rat liver injury and g) tail amputation models. Blood loss and hemostatic time in the h-i) rat liver injury and j-k) tail amputation models. The data are presented as the mean ± standard deviation, and statistical differences are expressed as *∗p < 0.05* and *∗∗p < 0.01*.

Due to the direct contact of CMF-CDs with wounds, favorable biocompatibility of CMF-CDs is essential. To assess the cytocompatibility of the CMF-CDs, RAW 264.7 cells and HUVECs were exposed to CMF-CDs for 24 h (Fig. 3a and b). Consequently, all groups presented high cell viability, surpassing 95 %. Moreover, the live/dead cell staining assay also revealed more than 90 % live cells (green fluorescence) and few dead cells (red fluorescence) after exposure to CMF-CDs, confirming their excellent cytocompatibility (Fig. 3d). Furthermore, a hemolytic test was performed to verify the hemocompatibility of the CMF-CDs. All tested concentrations of CMF-CDs showed remarkable hemocompatibility, as evidenced by a hemolysis ratio below 2 % (Fig. 3c). To evaluate the in vivo safety of CMF-CDs, we conducted HE staining of major organs after the CMF-CDs treatment. As a result, heart, liver, spleen, lung and kidney of the mice showed negligible damage between groups (Fig. S2), suggesting that CMF-CDs elicited high safety in vivo. Overall, above data indicated that CMF-CDs had biocompatibility, which provides the subsequent conduct for further in vivo application and future clinical trials.

In addition, because of the critical importance of cell migration in wound healing, we performed a RAW 264.7 cells scratch assay. As displayed in Fig. 3e, the CMF-CDs greatly enhanced cell migration in a dose-dependent manner. Compared with untreated cells with healing rate of 8.33 %, the scratch healing rate of RAW 264.7 cells treated with 0.25 mg/mL CMF-CDs for 24 h was approximately 40 %, suggesting the pro-healing activity of CMF-CDs in vitro (Fig. S3). Hemostasis, as the initial stage of wound repair, plays a crucial role in establishing a temporary barrier to protect the underlying tissue from additional damage and potential infection [43]. Considering the frequency and urgency of hemorrhagic wounds, an ideal wound dressing should have rapid and effective hemostasis properties. Charcoal drug-derived CDs have gained much attention as promising hemostatic dressings to effectively accelerate hemostasis by promoting platelet activation and coagulation pathways [5]. Therefore, we assessed the influence of CMF-CDs on hemostatic activity in vivo in a rat liver injury and tail amputation model. For the liver injury model, a wound 10 mm long and 3 mm deep in length was constructed in the liver and promptly sealed with CMF-CDs. As a result, we found that the CMF-CDs obviously bled less than the CMF-CDs in the control group and dextrin group (Fig. 3f). Dextrin is a soluble powder that has no hemostatic activity and is often used as the main ingredient in placebos in clinical trials. YunNanBaiYao (YNBY) consisted of *Panax notoginseng* (Sanqi in Chinese), *Aconiti Kusnezoffii Radix* (Caowu in Chinese) and *Rhizoma Paridis* (Chonglou in Chinese), is a classical Chinese medicine prescription and approved by the State Food and Drug Administration [5]. Owing to its pharmaceutical effects of hemostasis, anti-inflammatory and promoting wound healing, YNBY has been widely used to treat hemorrhagic diseases caused by various factors, including trauma, surgery and bruising Therefore, YNBY was considered as the positive drug to investigate the hemostatic properties of CMF-CDs. Further, the results of the quantitative analysis of blood loss and hemostatic time are presented in Fig. 3h and i, respectively. The control group with no treatment and dextrin group showed severe bleeding. After applying YNBY to the wound sites, the markedly decreased blood loss of 0.9 ± 0.3 g and a hemostatic time of 48 ± 20 s were observed. In particular, CMF-CDs showed similar effects to those of YNBY, with a blood loss of 1.0 ± 0.3 g and hemostatic time of 54 ± 10 s. H&E staining was performed to further understand the pathological states of the liver wounds. As shown in Fig. S4, massive amounts of blood cells and inflammatory cells were observed around the liver injury wounds in the control group. Nevertheless, this situation improved significantly after CMF-CD treatment. Moreover, for the rat tail amputation model, a 7.5 cm tail was cut off to further verify the rapid and effective hemostasis of the CMF-CDs (Fig. 3g). Similarly, the bleeding site was immediately sealed upon application of CMF-CDs, resulting in a substantial reduction in blood loss and hemostatic time (Fig. 3j and k). Together, CMF-CDs showed excellent hemostasis activity similar to those of YNBY, suggesting its significant value as a hemostatic and wound healing drug.

Chinese medicines have a great deal of active components and elements, making them rich in abundant amino, hydroxyl and carboxyl groups. CDs prepared by high-temperature pyrolysis using charcoal drugs endow them with wide biological activities. In this work, by using CMF as precursors, CDs with high water solubility and good biocompatibility can be easily acquired. Both a rat liver injury model and a tail amputation model have demonstrated the fast and effective hemostasis of CMF-CDs. CDs are widely applied in hemostasis therapy owing to the characteristics of charcoal drugs. The hemostatic activity may be attributed to the increased adsorption and convergence of CDs produced after carbonization of Chinese medicine. Many loose holes are formed in CDs, leading to physical adsorption and accelerating hemostasis. In addition, due to the specific structure of the CDs surface, it can activate plasma clotting factors to stop bleeding [34]. Currently, the clinical treatment of hemorrhagic diseases mainly depends on thrombin. However, thrombin, belonging to one type of proteins, is perishable and not conducive to long-term storage. Compared with proteins, CDs are more stable and more suitable [34]. Excessive ·OH, ·O_2_^−^ and H_2_O_2_ significantly increase the level of ROS in the wound, which induces oxidative stress in the wound and aggravates wound inflammation [44]. However, persistent inflammation is one of the main reasons for delayed wound repair [45]. Therefore, nanozymes with SOD-like or CAT-like activities can be used to reduce ROS in wounds to alleviate inflammation and promote repair [46]. To date, an increasing attention has been given to CDs nanozymes derived natural Chinese medicines, which shows a great potential as an antioxidant to remove excess ROS on wound surfaces [1]. Nevertheless, the development of multifunctional CDs for hemorrhagic wounds using natural biomass materials is a major challenge in current CDs-related researches. CMF, a special charcoal drug derived from fruit, has been used clinically to stop bleeding. In this study, CMF-CDs derived from CMF with high biocompatibility, exhibited SOD, CAT and GPx catalytic performance and strong ROS scavenging ability. At present, few studies on CDs-derived from charcoal drugs have focused on the activity of nanozymes. This study provides a novel strategy to develop therapeutic agents with hemostasis, ROS scavenging and pro-healing from charcoal drugs and natural products. Overall, charcoal drug-derived CDs could serve as alternative therapeutic agents for bleeding from trauma and healing of hemorrhage wounds.

### 2.4 *In vivo* dermal wound healing performance of CMF-CDs

**Fig. 4:**
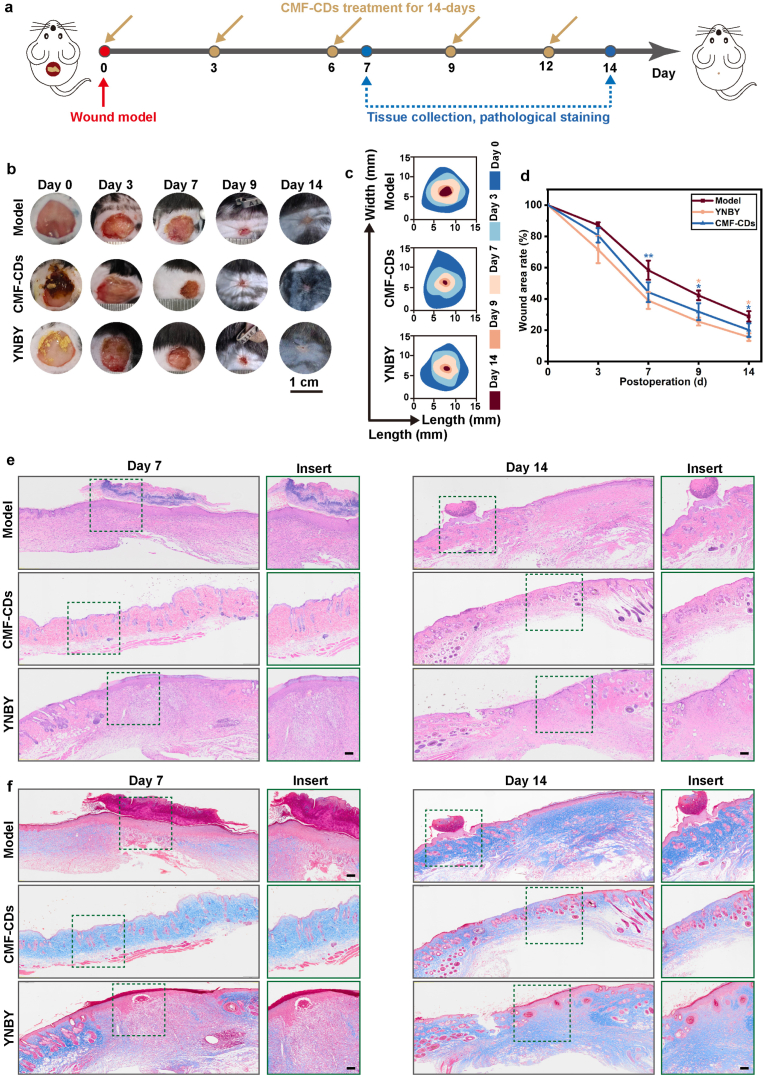
*In vivo* assessment of the effect of CMF-CDs on hemorrhagic wound healing. a) Diagram of the establishment and treatment of hemorrhagic wounds. b) Representative images of wounds and the dynamic repair process within 14 days. c) Traces of the wound repair process from days 0–14. d) Quantitative analysis of the wound area rate over time (n = 3). e) H&E-stained and f) Masson-stained images of regenerated skin tissue on days 7 and 14 (scale bar, 100 μm). The data are presented as the mean ± standard deviation, and statistical differences are expressed as *∗p < 0.05* and *∗∗p < 0.01*.

Encouraged by the excellent ROS scavenging performance, cell healing ability and favorable hemostatic activity of the biocompatible CMF-CDs, the practical efficacy of the CMF-CDs on hemorrhagic wounds was investigated. In this study, YNBY was selected to verify the wound healing activity of CMF-CDs in vivo. As illustrated in Fig. 4a, excisional skin wound models were constructed in C57BL/6 mice and treated with PBS (Model group), CMF-CD powder (CMF-CD group) or YNBY powder (YNBY group). The wounds were recorded and photographed every three days during the 14 days of treatment. Impressively, Notably, the wound healing rate in the CMF-CD treatment group was faster than that in the Model group and similar to that in the YNBY group (Fig. 4b). Furthermore, the dynamic process of wound healing with different treatments was simulated with a control map (Fig. 4c), and the wound area rate was calculated (Fig. 4d). Compared with that in model group, the wound area of mice in YNBY group and CMF-CD group was obviously recovered during the 14 days’ treatment. Specifically, the wound area of the CMF-CD group quickly decreased to (38.98 ± 5.37) % on day 7 and reached (15.58 ± 2.40) % on day 14, whereas the wounds in Model group remained unhealed. Moreover, the wound area of the YNBY group decreased from (44.32 ± 6.36) % to (20.10 ± 4.32) % with the increasing healing time from day 7 to day 14. In addition, during the whole healing process, there were no obvious difference of wound area of mice treated with YNBY and CMF-CDs. Notably, by day 14, the wounds treated with CMF-CDs were surrounded by abundant regenerated hair and showed remarkable wound healing performance, which was likely the result of the reshaping of the inflammatory microenvironment by the CMF-CDs.

Next, to evaluate tissue regeneration during wound healing, H&E and Masson staining were carried out. On day 7, the wounds in the Model and YNBY groups still exhibited evident hemorrhage and inflammatory tissue. However, fewer blood cells and inflammatory cells were observed in the CMF-CD group. Moreover, the thickness of mature granulation tissue is an important indicator to assess the wound repair process. As shown in Fig. 4e, intact epidermis grew after 14 days of CMF-CD treatment. Specifically, the thickness of mature granulation tissue in CMF-CD group was significantly larger than that in other treatment groups (Fig. S5). During the remodeling phase, proper collagen deposition and remodeling are crucial for improving the tensile strength of skin tissue and accelerating healing [3]. The high levels of ROS accumulation can hinder collagen fiber synthesis. Therefore, collagen deposition was measured by histological analysis on day 7 and 14. Masson staining results suggested that CMF-CDs markedly increased collagen deposition compared with that in the Model group, indicating the formation of new skin tissues (Fig. 4f and S6). On day 14, residual blood scabs were still observed in the Model group, indicating a delayed wound healing process. In comparison, more epithelial tissue and hair follicles adjacent to sebaceous glands were generated after CMF-CD treatment. Although the wound area rates of the CMF-CD group and YNBY group were almost the same, the skin tissue and hair follicle maturity of the CMF-CD group were remarkably greater than those of the YNBY group, suggesting that CMF-CDs have a greater ability to promote healing. These results thus demonstrated that CMF-CDs accelerated wound healing by enhancing rapid hemostasis, relieving inflammation, increasing collagen deposition and promoting the formation of mature epithelial structures.

### 2.5 Proteomic analysis of the therapeutic performance of CMF-CDs

**Fig. 5:**
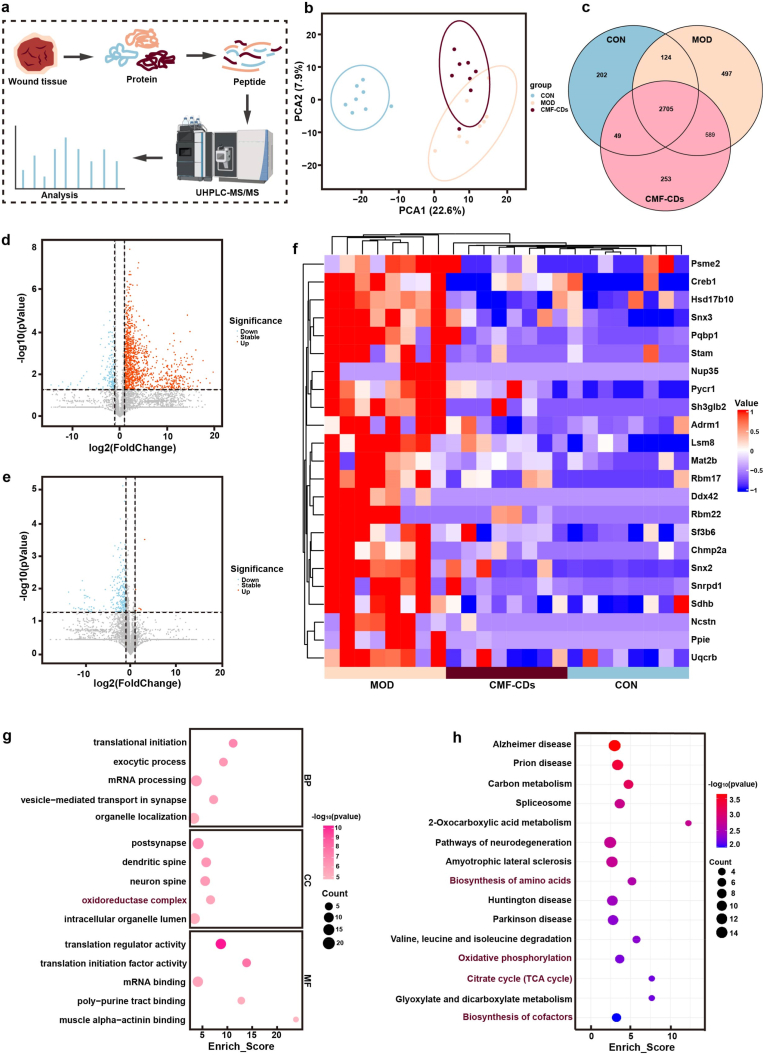
Proteomic analysis of hemorrhagic wound tissue after 14 days of CMF-CD treatment. a) Flow chart of the proteomics analysis. b) Principal component analysis (PCA) of the DEPs. Each data point corresponds to the PCA of each sample. c) Venn analysis of the proteins identified and quantified in the CON, MOD and CMF-CD groups. Volcano plots representing the downregulated and upregulated proteins between the d) MOD and CON groups and between the CMF-CDs and MOD groups. f) Heatmap of the common differentially expressed proteins between the MOD (n = 8), CMF-CD (n = 8) and CON groups (n = 8). g) GO enrichment and h) KEGG pathway analysis of the DEPs between the CMF-CD and MOD groups.

To elucidate the underlying mechanism of hemorrhagic wound healing after CMF-CD treatment, quantitative proteomics of skin wound tissues was performed (Fig. 5a). Principal component analysis (PCA) revealed substantially different proteomic profiles among the CON, MOD and CMF-CD groups (Fig. 5b). As shown in Fig. 5c, a Venn diagram revealed that 2705 proteins were expressed across all three sample groups, with unique expression of 202 proteins in the CON group, 497 proteins in the MOD group, and 253 proteins in the CMF-CD group. Furthermore, the differential protein expression levels are presented in volcano plots with the filter criteria of fold changes >1 or <1 and *p* < 0.05. As shown in Fig. 5d, among these differentially expressed proteins (DEPs), there were 1199 upregulated and 174 downregulated proteins in the MOD group compared with the CON group. Additionally, compared with those in the MOD group, 9 upregulated and 253 downregulated proteins were identified after CMF-CD treatment (Fig. 5e). Gene Ontology (GO) enrichment results of the differentially expressed proteins encompassed biological processes (BP), cellular components (CC) and molecular functions (MF). As shown in Fig. S7a, compared with those in normal skin, the results of the GO analysis of the upregulated DEPs after skin injury were mainly related to translation, ribonucleoprotein complex, and structural molecule activity. Furthermore, BP categorization indicated that the downregulated DEPs between the MOD and CMF-CD groups were mainly involved in translational initiation exocytic process, mRNA processing, vesicle-mediated transport in synapses and organelle localization. According to the MF analysis, these DEPs were mainly associated with translation regulator activity, translation initiation factor activity, mRNA binding and poly-purine tract binding (Fig. 5g). Notably, CC analysis confirmed that the downregulated DEPs were primarily located in intracellular organelles, especially the oxidoreductase complex. Comparison of the MOD and CON groups via Kyoto Encyclopedia of Genes and Genomes (KEGG) pathway enrichment results offered insights into changes in metabolic pathways such as ATP-dependent chromatin remodeling and neutrophil extracellular trap formation (Fig. S7b). After CMF-CD treatment, the KEGG pathways of the downregulated DEPs were mainly enriched in the citrate cycle (TCA cycle); oxidative phosphorylation (OXPHOS); valine, leucine and isoleucine degradation; glyoxylate and dicarboxylate metabolism; and the biosynthesis of cofactors (Fig. 5h). Among the top 15 signaling pathways, OXPHOS and the TCA cycle, which are known for their roles in mitochondrial dysfunction, were notably inhibited by CMF-CDs, as demonstrated in Fig. 5h. Mitochondria are essential organelles in eukaryotic cells that are essential in the highly metabolic wound repair process and are responsible for producing adenosine triphosphate (ATP) molecules through OXPHOS and the TCA cycle [47]. Apart from the basic energy supply, mitochondria are indispensable for regulating many metabolic processes such as phospholipid biosynthesis, calcium signaling and amino acid metabolism [48]. In the later stages of wound healing, mitochondria become longer and bifurcate, which is generally related to enhanced OXPHOS activity. Nevertheless, the enhanced fission activity in the earlier stages of wound healing subsequently affects mitochondrial function and contributes to increased ROS levels [49]. Therefore, mitochondrial integrity plays a crucial role in cell homeostasis and survival. In particular, the production of ATP through OXPHOS is accompanied by the inevitable generation of ROS, especially superoxide anion (·O_2_^−^), which has been implicated in pathological wound responses [49]. Normal amounts of ·O_2_^−^ can be converted to more stable hydrogen peroxide by natural SOD, which is essential for normal cellular signaling [50]. Nevertheless, the accumulation of excessive ·O_2_^−^ may trigger calcium (Ca^2+^) reflux and the release of cytochrome C to induce cell death. Additionally, the oxidative stress caused by excessive ROS release alters membrane permeability, resulting in delayed wound healing [51]. Thus, wound-induced oxidative stress results in mitochondrial dysfunction, and targeted clearance of ROS could serve as a great potential treatment to promote wound repair. Our results demonstrated that CMF-CDs with multienzyme-like activity could scavenge excessive ROS and regulate mitochondrial dysfunction by inhibiting OXPHOS and the TCA cycle, thereby accelerating wound healing.

Fig. 5f shows a heatmap showing the expression patterns of the 23 differentially expressed proteins between the MOD, CON and CMF-CD groups. Among these genes, SDHB protein expression was significantly downregulated after CMF-CD treatment, and the highest Log_2_FC was detected (Fig. S7c). As previously reported, mitochondria produce ATP by OXPHOS and the TCA cycle through the electron transport chain (ETC) [52]. Among the ETC components, succinate dehydrogenase (SDH) is essential to provide a biochemical link to OXPHOS and the TCA cycle [53]. SDH complex B (SDHB), a member of the SDH family, converts succinate to fumarate and participates in ETC and ATP production. The elimination of SDHB promotes the glycolytic energy metabolism, resulting in the Warburg effect and improving cell survival [54]. Conversely, the overexpression of SDHB prevents energy metabolism and hinders cell proliferation and vitality. It has been reported that SDH drives macrophage inflammation through disturbing mitochondrial metabolic reprogramming and specifically upregulates IL-1β expression [55]. Upon inhibiting succinic acid oxidation, the expression of IL-1β decreases in SDHB-deficient macrophages, thereby preventing the development of a proinflammatory phenotype [54]. Therefore, increased succinate oxidation by SDH in mitochondria is necessary for the induction of proinflammatory genes while limiting the induction of anti-inflammatory genes [55]. Above results revealed that downregulated expression of SDHB was observed after CMF-CD treatment, indicating reduced succinate oxidation in mitochondria and a proinflammatory phenotype in macrophages. According to the proteomic data, the in-depth mechanisms by which CMF-CDs promote wound healing were closely related to modulating mitochondrial dysfunction by countering OXPHOS and the TCA cycle, scavenging excessive ROS and inhibiting the proinflammatory macrophage phenotype, thereby reshaping the inflammatory microenvironment.

### 2.6 Regulation of the inflammatory microenvironment by CMF-CDs

**Fig. 6:**
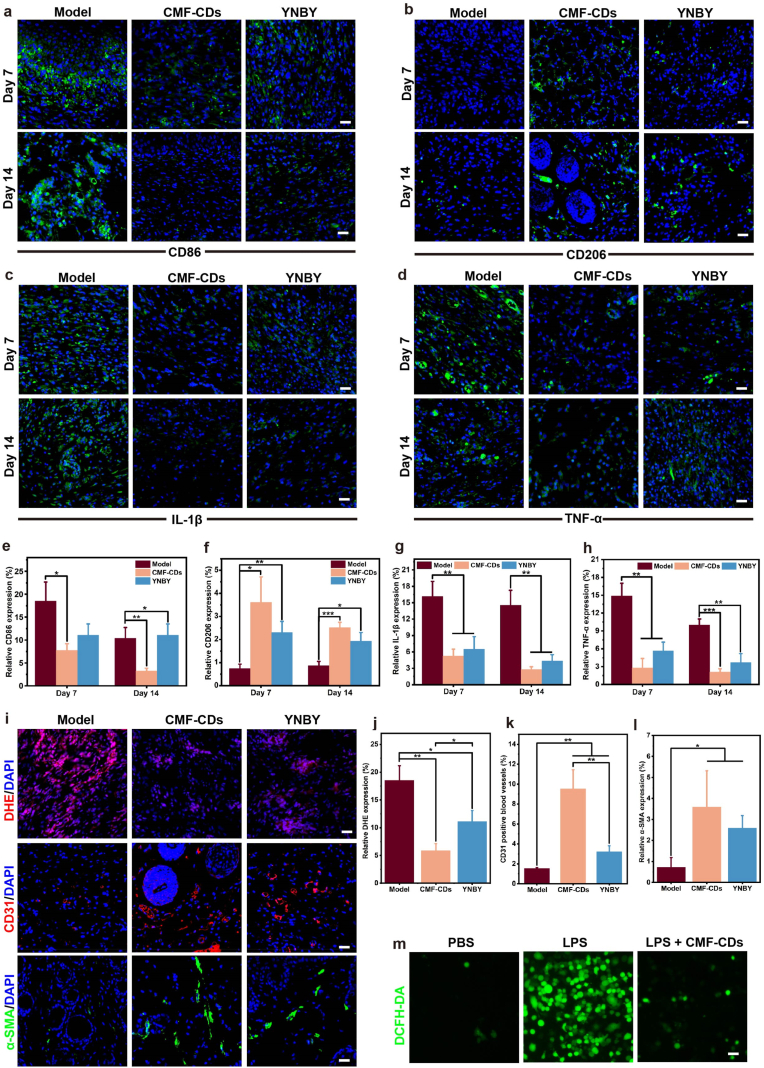
Inflammatory regulation effects of CMF-CDs in vivo and in vitro. Immunofluorescence staining of a) CD86, b) CD206, c) IL-1β, and d) TNF-α expression in wound tissues on day 7 and day 14 (scale bar, 100 μm). Quantitative analysis of immunofluorescence staining of e) CD86, f) CD206, g) IL-1β and h) TNF-α in wound tissues after CMF-CD treatment (n = 3). i) Immunofluorescence staining of DHE, CD31 and α-SMA expressions in wound tissues on day 14 (scale bar, 100 μm). Quantitative analysis of immunofluorescence staining of j) DHE, k) CD31 and l) α-SMA and expressions in wound tissues after CMF-CD treatment (n = 3). m) Fluorescence imaging of intracellular ROS scavenging in RAW264.7 cells treated with CMF-CDs (scale bar, 100 μm).

Wound repair is a choreographed biological process involving hemostasis, anti-inflammation, angiogenesis, extracellular matrix remodeling and re-epithelialization. During wound healing, macrophages play an essential role in matrix remodeling and tissue repair. The endogenous antioxidant system may be impaired by metabolic alterations, as characterized by the impaired transition of macrophages from a “pro-inflammatory” (M1) phenotype to a “pro-healing” (M2) phenotype, resulting in a persistent inflammatory microenvironment [8]. Therefore, the macrophage phenotype from the M1 to M2 phenotype could establish an anti-inflammatory microenvironment to achieve ideal wound repair. To further understand the effect of CMF-CDs on macrophage phenotypes in skin wounds, the expression levels of CD86 (M1-type marker) and CD206 (M2-type marker) were visualized by immunofluorescence staining. As a result, the high proportion of M1 phenotype macrophage was observed in the Model group during the healing process, suggesting a severe and persistent inflammatory response (Fig. 6a and b). On day 7, in comparison with those in the Model group, an increased proportion of M2 phenotype macrophages and a decreased proportion of M1 phenotype macrophages were observed after CMF-CD treatment, accompanied by reduced secretion of proinflammatory cytokines such as TNF-α, IL-6 and IL-1β (Fig. 6c and d and Fig. S8). By day 14, the wounds in the Model group still exhibited abundant M1 macrophages and proinflammatory cytokines, which contributed to delayed wound healing. Nevertheless, CMF-CD treatment markedly decreased the ratio of proinflammatory macrophages to proinflammatory cytokines on day 14. Further, on day 7 and 14, the expressions of CD86, CD206, TNF-α, and IL-1β in different treated wound tissues was quantified (Fig. 6e-h).

Preventing excessive accumulation of ROS in the early stage of wound contributes to reduce inflammatory response and accelerate wound repair [1]. Dihydroethidium (DHE) staining was performed to assess the ROS accumulation in skin wound tissues with different treatments. As shown in Fig. 6i and j, ROS levels were significantly lower in CMF-CDs-treated skin wounds than in untreated skin wounds. Additionally, compared with YNBY treatment, CMF-CDs showed stronger ROS scavenging effect in wound tissue, which might be ascribed to the enzymatic catalytic activities of CDs. During wound repair, inhibiting the inflammatory response promotes the neovascularization regeneration, provides oxygen and nutrients, and facilitates the tissue remodeling [56]. Hence, angiogenesis is of a great significance for wound repair and skin regeneration. To explore neovascularization in skin wounds, CD31 and α-SMA immunofluorescence staining was performed. As shown in Fig. 6i, k and 6l, CMF-CD treatment markedly increased the expression of CD31 and α-SMA in regenerated skin on day 14, suggesting strong neovascularization. Consequently, the enhancement of neovascularization facilitates the transport of nutrients and oxygen to support cell proliferation and tissue reconstruction during wound healing [57]. Consistent with the proteomic analysis results, CMF-CDs effectively driven the transition of macrophages from the M1 to M2 phenotype, decreased the ROS levels, subsequently modulating the inflammatory microenvironment and enhancing neovascularization, thereby contributing to the efficient healing of hemorrhagic wounds in vivo.

Oxidative stress refers to the imbalance of the body's oxidation and antioxidant systems, and oxidation tends to produce a large number of ROS intermediates [18]. ROS is critical for cell homeostasis and intercellular communication; however, excessive ROS can damage cells, inhibit angiogenesis, trigger inflammation and oxidative stress, and hinder the wound repair process [58]. Given the multienzyme-like ability of CMF-CDs to scavenge ROS, the intracellular antioxidant capacity and cytoprotective effect of CMF-CDs were further investigated. As shown in Fig. 6m, in the presence of lipopolysaccharide (LPS), large amounts of ROS generated in RAW 264.7 cells were visualized by the 2′,7′-dichlorodihydrofluorescein diacetate (DCFH-DA) probe. Such excess ROS were effectively eliminated by CMF-CDs at a concentration of 250 μg/mL. Furthermore, the cytoprotective effect of CMF-CDs on RAW 264.7 cells upon LPS exposure were investigated. The live/dead cell staining assay demonstrated that LPS stimulation significantly induced cell death (PI-positive cells with red fluorescence). However, cell death induced by LPS was essentially abrogated by CMF-CDs (Fig. S9). The above results suggested that CMF-CDs with excellent multienzyme-like antioxidant activity can protect cells from oxidative stress-induced damage by eliminating intracellular ROS. Recently, CD nanozymes have gained remarkable attention for their application in wound healing. The present study revealed that CMF-CDs alleviated the inflammatory microenvironment by regulating the macrophage phenotype, scavenging excessive ROS, reducing proinflammatory cytokines, promoting collagen deposition, and facilitating angiogenesis and follicular regeneration, thereby accelerating wound repair. This helps build scar-free, physiologically normal regenerated skin and accelerates wound repair.

## 3 Conclusion

In summary, CDs from natural CMF were developed to rescue hemorrhagic wound healing. Due to the strong oxidative free radical scavenging abilities, the CMF-CDs proficiently minimized high intracellular ROS levels. In particular, the CMF-CDs rapidly and effectively promoted hemostatic effects. *The* in vivo hemorrhagic wound treatment results further confirmed the remarkable anti-inflammatory-assisted vascularization, collagen formation and follicle regeneration capabilities of the CMF-CDs, which contributed to enhanced wound healing outcomes. Proteomic assessment demonstrated that CMF-CDs can reshape the inflammatory microenvironment by modulating OXPHOS and TCA cycle metabolism. Overall, CMF-CDs with outstanding hemostasis and ROS-scavenging properties have great potential as promising candidates for treating hemorrhagic and inflammation-related diseases. By combining charcoal drug with modern nanotechnology, our findings provide a promising paradigm for all-in-one therapy.

## CRediT authorship contribution statement

**Pan Liang:** Writing – original draft, Funding acquisition, Formal analysis, Conceptualization. **Yining Ma:** Methodology, Formal analysis, Data curation. **Menglin Song:** Formal analysis, Data curation. **Hong Wang:** Methodology, Formal analysis. **Xin Peng:** Methodology, Investigation. **Qin Sun:** Investigation, Data curation. **Hongping Shen:** Resources, Methodology. **Zengjin Liu:** Supervision, Software. **Pei Luo:** Writing – review & editing, Funding acquisition, Formal analysis, Data curation. **Wei Ren:** Writing – review & editing, Funding acquisition, Formal analysis, Data curation.

## Declaration of competing interest

We declared that we have no conflicts of interest to this work.

We declare that we do not have any commercial or associative interest that represents a conflict of interest in connection with the work submitted.

## Data Availability

Data will be made available on request.
