# Supplementary material for: Design of nano mume Fructus charcoal by reshaping inflammatory microenvironment as biofunctional wound nanomedicine
Source: Mater Today Bio. 2025 Jun 9;33:101971. doi: 10.1016/j.mtbio.2025.101971 (PMC12205667; doi:10.1016/j.mtbio.2025.101971)
Supplement: Multimedia component 1 [file mmc1.docx]

**Supporting Information**

**Design of nano Mume Fructus charcoal by reshaping inflammatory microenvironment as biofunctional wound nanomedicine**

***Pan Liang^1,2,#,*^, Yining Ma^1,#^, Menglin Song^1,#^, Hong Wang^1^, Xin Peng^1^*, *Qin Sun^1^, Hongping Shen^1^, Zengjin Liu^1^*, *Pei Luo^2,*^*, *Wei Ren^1,*^***

**^1^** Drug Research Center of Integrated Traditional Chinese and Western Medicine, the Affiliated Traditional Chinese Medicine Hospital, Southwest Medical University, Luzhou 646000, China.

**^2^** State Key Laboratories for Quality Research in Chinese Medicines, Faculty of Chinese Medicine, Macau University of Science and Technology, Macau 853, China.

**^#^** These authors contributed equally to this work and are considered co-first authors.

*** Correspondence:**

*Dr. Associate Prof. Wei Ren,*

Drug Research Center of Integrated Traditional Chinese and Western Medicine, the Affiliated Traditional Chinese Medicine Hospital, Southwest Medical University, Luzhou 646000, China.

E-mail address: renwei1991@swmu.edu.cn (W. Ren)

*Dr. Prof. Pei Luo*,

State Key Laboratories for Quality Research in Chinese Medicines, Macau University of Science and Technology, Macau 999078, China.

E-mail address: [pluo@must.edu.mo](mailto:pluo@must.edu.mo) (P. Luo)

*Dr. Pan Liang,*

Drug Research Center of Integrated Traditional Chinese and Western Medicine, the Affiliated Traditional Chinese Medicine Hospital, Southwest Medical University, Luzhou 646000, China.

E-mail address: [xnydzyylp@swmu.edu.cn](mailto:xnydzyylp@swmu.edu.cn) (P. Liang)

**Coauthor e-mail addresses**: [xnydzyylp@swmu.edu.cn](mailto:xnydzyylp@swmu.edu.cn) (P. Liang), [20210399120027@stu.swmu.edu.cn](mailto:20210399120027@stu.swmu.edu.cn) (Y. Ma), 20220399120035@stu.swmu.edu.cn (M. Song), 20230399120020@stu.swmu.edu.cn (H. Wang), 20220314330714@stu.swmu.edu.cn (X. Peng),  [zxyjhsq@swmu.edu.cn (Q. Sun),](mailto:15082068899@163.com,) [15082068899@163.com](mailto:15082068899@163.com) (H. Shen), zengjinliu@swmu.edu.cn (Z. Liu), [pluo@must.edu.mo](mailto:pluo@must.edu.mo) (P. Luo), renwei1991@swmu.edu.cn (W. Ren).

**Experiments section**

**Chemicals and Materials**

CMF was provided by Baicaotang Herbal decoction Pieces Co., LTD (Luzhou, China, batch number: 220307-1). D_2_O (99.9% D) was purchased from Adamas Beta (Shanghai, China). Dialysis bags (MWCO: 1000 Da) were obtained from Solarbio Science & Technology Co., Ltd. (Beijing, China). 2,2-Biphenyl-1-picrohydrazyl (DPPH) was purchased by ACMEC Biochemical Co., Ltd. (Shanghai, China). 2,2-Dimethyl-3,4-dihydro-2H-pyrrole 1-oxide (DMPO) was provided by Glpbio (Montclair, CA, USA). A glutathione peroxidase (GSH-Px) assay kit and a hydrogen peroxide detection kit were provided by Nanjing Jiancheng Bioengineering Institute (Nanjing, China). Acetic acid/sodium acetate buffer solution (HAc/NaAc, 0.1 mol/L, pH = 4.5), hydrogen peroxide solution (H_2_O_2_, 3 g/100 mL) and the total antioxidant capacity assay kit were purchased from Yuanye Bio-Technology Co., Ltd. (Shanghai, China). L-Methionine, riboflavin, nitrotetrazolium blue chloride (NBT) and lipopolysaccharide (LPS) were provided by Aladdin Industrial Corporation (Shanghai, China). Superoxide dismutase (SOD) assay kit was provided by Dojindo Molecular Technologies (Japan). Terephthalic acid (TPA) and 3,3’,5,5’-Tetramethylbenzidine (TMB) were provided by Beyotime Biotechnology Co., Ltd. (Shanghai, China) and Macklin (Shanghai, China), respectively. DCFH-DA probe was provided by MedChemExpress (New Jersey, USA).

**Cells and animals**

Mouse leukemia monocyte macrophages (RAW 264.7) and human umbilical vein endothelial cells **(**HUVECs) were provided by iCell Bioscience Inc. (Shanghai, China). Male C57BL/6 mice (22 ± 2 g) and male Sprague‒Dawley (SD) rats (weight: 200-220 g) were provided by GemPharmatech Co., Ltd. (Chengdu, China). Animal welfare and experimental procedures are carried out in strict accordance with the “Guidelines for the Care and Use of Experimental Animals” and approved by the Animal Care and Use Committee of Southwest Medical University (Luzhou, China, #20231218-017).

**Synthesis of CMF-CDs**

A total of 20 g of CMF powder was soaked and boiled in 600 mL of deionized water for 1 h. After cooling, the resulting solution was coarsely filtered, fine filtered and concentrated. Then, the resulted product was purified using a dialysis bag (MWCO: 1000 Da) to remove chemical components. Finally, the CMF-CDs were lyophilized to obtain powders and stored at 4 ℃ for further characterization.

**Characterization of CMF-CDs**

High-resolution transmission electron microscopy (HR-TEM, JEM 2100 F, JEOL, Japan) was applied to visualize the morphology of the CMF-CDs. The structural properties of the CMF-CDs were determined using an X-ray diffractometer (Ultima IV, Rigaku, Japan) and a confocal Raman microspectrometer (LabRAM HR Evolution, Horiba Scientific, France). A double-beam UV‒vis spectrophotometer (TU-1900, Shanghai, China) and fluorescence spectrophotometer (FS5, Edinburgh, UK) were used to investigate the absorption and fluorescence spectra of the CMF-CDs, respectively. The Fourier transform infrared spectroscopy (FT-IR) spectrum of the CMF-CDs was performed using an infrared spectrometer (IRTracer 100, Shimadzu, Japan). The elemental composition of the CMF-CDs was determined via X-ray photoelectron spectroscopy (XPS, Kratos/AXIS SUPRA+, Shimadzu, Japan). Chemical components of CMFs and CMF-CDs was analyzed using a UHPLC System (ThermoFisher, UltiMate3000) connected to an Orbitrap mass spectrometer (ThermoFisher, Q Exactive). A Bruker AVANCE III HD spectrometer was used to record the ^1^H-nuclear magnetic resonance (^1^H-NMR) spectra of the CMF-CDs at 400 MHz in D_2_O.

**In vitro and in vivo stability of CMF-CDs**

The ζ-potential changes of CMF-CDs during storage can reflect its stability. Detailly, 1 mg/mL CMF-CDs solution in pH = 7.4 PBS and pH = 7.4 PBS containing 10% fetal bovine serum (FBS) were prepared, respectively. The ζ-potential of CMF-CDs was measured at 0, 1, 3, 5, 7 and 14 days using nano particle size analyzer (90plus PALS, Bruker, Germany).

**·OH scavenging ability of the CMF-CDs**

The ·OH scavenging capability was tested by the 3,3’,5,’-tetramethylbenzidine (TMB) chromogenic method. ·OH was generated by H_2_O_2_ and Fe^2+^ through the classical Fenton reaction, thereby forming the oxidized TMB (oxTMB) with a distinct absorption at 652 nm.[1] Therefore. The reaction system solution was prepared with 0.5 M pH = 4.5 HAc/NaAc buffer containing 2 mM H_2_O_2_, 1 mM FeSO_4_, 250 μM TMB and various concentrations of CMF-CDs (0.0039-1 mg/mL) and incubated for 5 min. The reaction system solution without CMF-CDs was used as a positive control. Finally, the characteristic absorbance at 652 nm was determined via UV–vis spectroscopy and a microplate reader, and the ·OH scavenging ability of the CMF-CDs was calculated.

·OH scavenging rate (%) = (A_1_ – A_0_)/(A_2_ – A_0_) × 100%

In the above formula, A_1_, A_2_, and A_0_ correspond to the absorbance of the supernatant treated with CMF-CDs, the positive control group and the blank group at 652 nm, respectively.

**·O_2_^-^ scavenging ability of the CMF-CDs**

The ·O_2_^-^ scavenging ability of the CMF-CDs was determined by measuring the photoreduction inhibition rate of nitrotetrazolium blue chloride (NBT)[2]. The reaction solutions were prepared with 25 mM pH = 7.4 PBS buffer containing 20 μM riboflavin, 50 μM NBT, 13 mM L-methionine and various concentrations of CMF-CDs (0.0039-1 mg/mL). After the 5 min of LED irradiation, the absorbance of the reaction solutions at 560 nm was detected by UV–vis spectroscopy and a microplate reader. Samples containing riboflavin, L-methionine and NBT after LED irradiation were used as positive controls. The ·O_2_^-^ scavenging rate was calculated as follows:

·O_2_^-^ scavenging rate (%) = (A_1_ – A_3_)/(A_2_ – A_3_) × 100%

In the above formula, A_1_, A_2_, and A_3_ correspond to the absorbance of the supernatant treated with CMF-CDs, the positive control group and the blank group at 560 nm, respectively.

**Electron spin resonance (ESR) Assay**

5,5-Dimethyl-1-pyrroline-N-oxide (DMPO) was used to trapping ·OH or ·O_2_^-^. Specifically, to test ·OH scavenging activity, 0.25, 0.5 and 1 mg/mL CMF-CDs were added to 0.5 M pH = 4.5 HAc/NaAc buffer containing 2 mM H_2_O_2_, 1 mM FeSO_4_, 250 μM TMB, and 20 mM DMPO, respectively. The ESR spectral signal was collected on an Elexsys E500 spectrometer (Bruker, Germany). To test the ·O_2_^-^ scavenging activity, 0.25, 0.5 and 1 mg/mL CMF-CDs were mixed with 25 mM pH= 7.4 PBS buffer containing 20 μM riboflavin, 13 mM L-methionine, 50 μM NBT and 20 mM DMPO, respectively. After LED irradiation for 5 min, the residual ·O_2_^-^ was recorded by ESR spectroscopy.

**H_2_O_2_ scavenging ability of the CMF-CDs**

The H_2_O_2_ scavenging ability of the CMF-CDs was evaluated with a hydrogen peroxide detection kit [1]. Briefly, H_2_O_2_ could form the yellow complex with ammonium molybdate, showing a characteristic peak at 405 nm. Numerous concentrations of CMF-CDs (0.0039-1 mg/mL) were added to 2 mM H_2_O_2_ at 37 °C. A reaction solution without CMF-CDs was selected as a positive control. After reacting for 2 h, the remaining H_2_O_2_ in the solution was detected according to the product instructions, and the H_2_O_2_ scavenging rate was calculated:

H_2_O_2_ scavenging rate (%) = (A_1_ – A_0_)/(A_2_ – A_0_) × 100%

In the above formula, A_1_, A_2_, and A_0_ correspond to the absorbance of the supernatant treated with CMF-CDs, the positive control and the blank control at 405 nm, respectively.

**Reactive nitrogen species** **(RNS) scavenging capability of the CMF-CDs**

The ability of the CMF-CDs to scavenge reactive nitrogen species was assessed using 2,2-diphenyl-1-pyridylhydrazine (DPPH·), which exhibited an obvious absorption peak at 515 nm.[3] Briefly, various concentrations of CMF-CDs (0.0039-1 mg/mL) were added to 125 μM DPPH· ethanol solution and incubated for 30 min. PBS was used for the control group. The characteristic absorbance at 515 nm was determined via UV–vis spectroscopy, and the DPPH· scavenging ability of the CMF-CDs was calculated as follows:

DPPH· scavenging rate (%) = (A_1_ – A_0_)/A_0_ × 100%

In the above formula, A_1_ and A_0_ represent the absorbances of the CMF-CDs group and control group at 515 nm, respectively.

**Total antioxidant capacity of the CMF-CDs**

The total antioxidant ability of the CMF-CDs was investigated by the 2,2’-azino-bis (3-ethylbenzthiazoline-6-sulfonic acid) (ABTS) method. Specifically, various concentrations of CMF-CDs (0.0039-1 mg/mL) were mixed with 2 mL of ABTS^+·^ radical solution and reacted for 5 min. PBS was used for the control group. Finally, the characteristic absorbance of samples at 734 nm was detected by UV–vis spectroscopy.

ABTS^+·^ scavenging rate (%) = (A_1_ – A_0_)/A_0_ × 100%

In the above formula, A_1_ and A_0_ represent the absorbances of the CMF-CD group and control group at 734 nm, respectively.

**SOD-like activity of CMF-CDs**

A commercial colorimetric SOD assay kit (WST-1 method) was applied to assess the SOD-like activity of the CMF-CDs.[4] ·O_2_^-^ was produced from the xanthine and xanthine oxidase system, thereby converting WST-1 to its formaldehyde with characteristic absorbance at 450 nm. Specifically, 0.0039-1 mg/mL of the CMF-CDs were mixed with 5 mM xanthine and 0.5 U/mL xanthine oxidase, and incubated at 37 °C for 10 min. The absorbance at 450 nm was determined by a multimode reader (SP-Max3500, Flash, Shanghai, China).

**CAT-like activity of CMF-CDs**

The CAT-like activity of the CMF-CDs was evaluated via fluorescence spectroscopy[1]. Briefly, terephthalic acid (TPA) without fluorescence, can react with the ·OH of H_2_O_2_ decomposition to form 2-hydroxyterephthalic acid (Excitation: 315 nm, Emission: 425 nm). CAT-like substances catalyze the decomposition of H_2_O_2_ into H_2_O and O_2_ and fail to generate 2-hydroxyterephthalic acid. Therefore, the CAT-like activity of the CMF-CDs was determined by monitoring the fluorescence signal using a fluorescence spectrophotometer (FS5, Edinburgh, UK).

**GPx-like activity of CMF-CDs**

A GPx assay kit was used to assess the GPx-like activity of the CMF-CDs. In this method, reducing glutathione (GSH) is oxidized to oxidizing glutathione (GSSG) and then reduced to GSH by glutathione reductase. Then, the concentration of reducing nicotinamide adenine dinucleotide phosphate (NADPH) decreased, which could be detected by UV–vis spectroscopy at 340 nm. The decrease in the NADPH concentration is directly proportional to the GPx-like activity of the CMF-CDs.

**Intracellular ROS scavenging ability of CMF-CDs**

The ability of the CMF-CDs to scavenge intracellular ROS was tested using an ROS assay kit. RAW 264.7 cells were seeded and cultured for 24 h. Then, the culture medium was replaced with various concentrations of the CMF-CDs for 24 h. After that, 1 µg/mL LPS was added for 24 h of incubation. Cells without added LPS or CMF-CDs were considered negative controls, while cells without added CMF-CDs were considered positive controls. Next, 10 μM DCFH-DA was added for another 30 min incubation. Finally, the fluorescence of the RAW 264.7 cells was visualized by a fluorescence microscope (ECLIPSE Ts2R, Nikon, Japan).

**Cytoprotective ability of CMF-CDs under oxidative stress**

The cytoprotective ability of CMF-CDs under oxidative stress conditions was investigated. Briefly, RAW 264.7 cells were seeded and cultured with DMEM containing 10% FBS. After that,1 µg/mL LPS was added for 24 h of incubation, followed by incubation with various concentrations of the CMF-CDs. Then, the cells were washed and stained with a live/dead cell staining kit. Finally, the fluorescence of the RAW 264.7 cells was analyzed by a fluorescence microscope (ECLIPSE Ts2R, Nikon, Japan) to visualize the live cells and dead cells.

**Cytocompatibility of the CMF-CDs**

A Cell Counting Kit-8 (CCK8) assay was carried out to investigate the cytocompatibility of the CMF-CDs. Briefly, RAW 264.7 cells or HUVECs were seeded and cultured with DMEM containing 10% FBS. After treatment with 200 μL of 0.0039-1 mg/mL CMF-CDs in blank DMEM for 24 h, the supernatant in each well was discarded, and the medium was replaced with 10% CCK-8 solution for another 2 h of incubation. Blank DMEM was used for the control group. Finally, the absorbance at 450 nm was detected through a multimode reader (SP-Max3500, Flash, Shanghai, China), and the cell viability (%) was calculated:

Cell viability (%) = (A_1_ – A_0_)/(A_2_ – A_0_) × 100%

In the above formula, A_1_, A_2_, and A_0_ correspond to the absorbance of the CMF-CDs, control group and blank group at 450 nm, respectively.

**Cell scratch healing ability of CMF-CDs**

A cell scratch experiment was performed to explore the impact of CMF-CDs on cell migration *in vitro*. RAW264.7 were seeded and cultured at a confluence of approximately 85%. A 200 µL pipette tip was applied to make a scratch in each well. The time point postscraping was marked as the 0-hour point. Next, the cells were incubated with various concentrations of the CMF-CDs for 24 h. The migrated cells were photographed by microscopy (Nikon Eclipse, TS2, Tokyo, Japan).

**Hemocompatibility of the CMF-CDs**

Fresh rat blood was collected to test the hemocompatibility of the CMF-CDs. Specially, 200 μL of blood was added with 800 μL of PBS containing different concentrations of CMF-CDs for 3 h of incubation at 37 °C. Pure PBS and Triton X-100 was selected as the negative and positive control, respectively. After centrifugation for 10 min, 100 μL of the supernatant was removed, and the absorbance at 540 nm was analyzed by a multimode reader (SP-Max3500, Flash, Shanghai, China). The hemolysis rate (%) was calculated:

Hemolysis rate (%) = (A_1_ – A_0_)/(A_2_ – A_0_) × 100%

In the above formula, A_1_, A_2_, and A_0_ correspond to the absorbance of the supernatant treated with CMF-CDs, Triton X-100 and PBS at 540 nm, respectively.

**In vivo safety of the CMF-CDs**

C57BL/6 mice were randomly divided into the Control group (without any treatments), CMF-CD group (treated with CMF-CD powder, 1 mg) and YNBY group (treated with Yunnan Baiyao powder, 1 mg). After 14 days of treatment, the major organs of all mice were isolated, fixed with 4% polyformaldehyde, and sectioned. Finally, pathological conditions of major organs were observed by H&E staining to evaluate in vivo safety of the CMF-CDs.

**Hemostasis and liver wound healing *in vivo***

A liver wound model was generated to evaluate the effective hemostasis of CMF-CDs *in vivo*. Briefly, male SD rats (200-220 g) were anesthetized, and the abdominal cavity was opened to expose the liver. After placing the filter paper under the liver, a wound (length: 10 mm, deep: 3 mm) was made to cause bleeding. A total of 40 mg of CMF-CDs or Yunnan Baiyao (YB) powder was applied to the bleeding site within 5 seconds, while untreated rats were used as a negative control. Bleeding loss (mg) and bleeding time (s) were recorded. Finally, the liver wounds were excised, fixed, embedded and sectioned, and pathological conditions were observed by H&E staining.

Bleeding loss (mg) = M_A_ – M_B_

In the above formula, M_B_ and M_A_ are the weights of the filter paper before and after absorbing blood, respectively.

**Hemostasis and tail wound healing *in vivo***

Similarly, a tail wound model was established to evaluate the effect of rapid hemostasis with CMF-CDs *in vivo*. Briefly, male SD rats (200-220 g) were anesthetized. Then, the tail was disinfected with 75% ethanol. After placing the filter paper under the tail, the 8 cm long tail was cut off to cause bleeding. CMF-CDs or Yunnan Baiyao (YNBY) powder was applied to the bleeding site within 5 seconds, while untreated rats were used as a negative control. Bleeding loss (mg) and bleeding time (s) were recorded.

Bleeding loss (mg) = M_A_ – M_B_

In the above formula, M_B_ and M_A_ are the weights of the filter paper before and after absorbing blood, respectively.

Bleeding time (s) = T_A_ – T_B_

In the above formula, T_A_ and T_B_ are the time at which the bleeding started and stopped, respectively.

***In vivo* hemorrhagic wound repair assessment**

A full-thickness cutaneous hemorrhagic wound model was built in C57BL/6 mice to verify the tissue repair ability of CMF-CDs. Typically, full-thickness cutaneous wounds with a 1 cm diameter on the dorsal skin were made using surgical scissors after anesthesia and hair removal. After that, all animals were randomly assigned into three groups: Model group (without any treatments), CMF-CD group (treated with CMF-CD powder, 1 mg) and YNBY group (treated with Yunnan Baiyao powder, 1 mg). Wound images were then recorded on days 0, 3, 7, 9 and 14 after treatment, and the wound size was analyzed by ImageJ software (NIH, USA).

**Proteomic analysis of skin wounds after CMF-CD treatment**

C57BL/6 mice were randomly assigned into the CON group (n = 8), the MOD group (n = 8), and the CMF-CD group (n = 8). After 14 days of treatment, the mice were sacrificed, and the cutaneous skin was collected. As previously reported, total protein extraction from skin tissues and enzymatic hydrolysis of peptides were performed.[5]

All samples were performed on a HPLC system (EASY-nLC 1200, Thermo Fisher Scientific, USA) using a Nano Chrom-C18 column (15 cm, ID150 μm, 1.9 μm, C18). The peptide samples were separated by an analytical chromatographic column and analyzed by a mass spectrometer (Q Exactive HF-X, Thermo Fisher Scientific, USA). The flow rate was 600 nL/min. Full MS scans in positive mode in the mass range of 300-1400 m/z were collected with a mass resolution of 120000 at 200 m/z. The maximum injection time was 30 ms, the automatic gain control target value was 3 × 10^6^, and the fusion dynamic exclusion was 12.0 s. The top ten intense MS peaks were fragmented with a collision energy of 27% and an isolation window of 1.6 m/z.

The original mass spectrometry data were generated from RAW files, and the qualitative and quantitative analysis was performed on iProteome cloud platform. For bioinformatics analysis, the expression levels of the target proteins were first normalized (z score). The global false discovery rate (FDR) was 0.01, and more than two peptides were required to quantify the protein groups. The differentially expressed proteins were identified using R statistical package software with the filter criteria of fold changes > 1 or < 1 and p < 0.05. Blast2GO (#BLASTP 2.8.0+) was used to analyze Gene Ontology (GO) enrichment analysis of targeted proteins. The differentially expressed proteins (DEPs) were remarkably enriched through Kyoto Encyclopedia of Genes and Genomes (KEGG) functional enrichment analysis.

**Investigation of wound healing mechanisms**

Mice were euthanized and dissected to retrieve regenerated back skin, which was collected at different time points and fixed. After being embedded in paraffin and sectioned, tissue slices were subjected to H&E, Masson, platelet endothelial cell adhesion molecule-1 (CD31), , tumor necrosis factor-alpha (TNF-𝛼), interleukin-6 (IL-6), CD206 (M2 indicator), CD86 (M1 indicator), interleukin-1β (IL-1β), and α-smooth muscle actin (α-SMA) staining. Finally, the slices were observed under a microscope (CIC, XSP-C204) to capture images.

**Statistical analysis**

The data are presented as the mean ± standard deviation (S.D.). Differences between two groups were evaluated using one-way analysis of variance (ANOVA). Specifically, *p* < 0.05 is considered to be significant difference, expressed by * (*p* < 0.05) and ** (*p* < 0.01). Figures and graphs were prepared with Origin 9.1, Adobe Photoshop 2020 and Adobe Illustrator 2023.

**Supplementary Figures section**

**
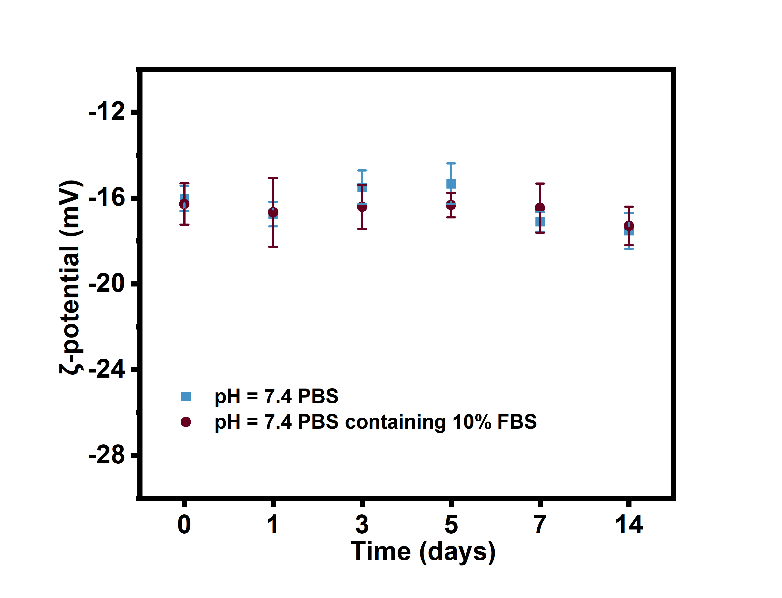
**

**Fig. S1.** ζ-potential of CMF-CDs during the 14 days’ storage in pH = 7.4 PBS and pH = 7.4 PBS containing 10%FBS (n = 3).

**
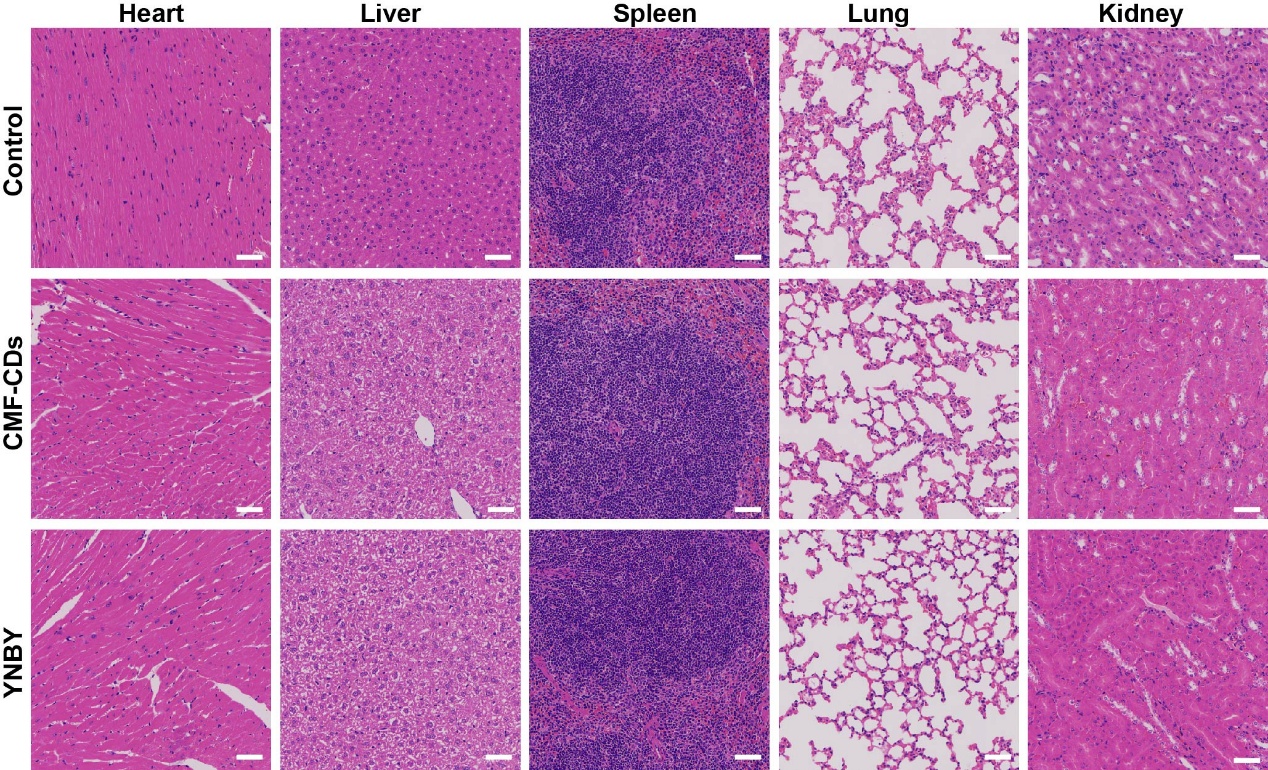
**

**Fig. S2.** HE staining of heart, liver, spleen, lung and kidney with different treatments (scale bar: 100 μm, n = 3).

**
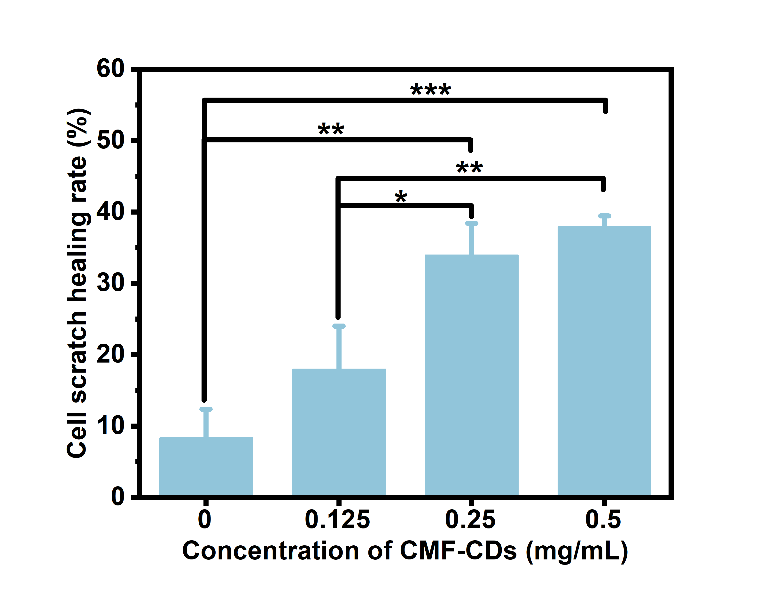
**

**Fig. S3.** The cell scratch healing rate of RAW 264.7 cells after treated with different concentration of CMF-CDs.

**
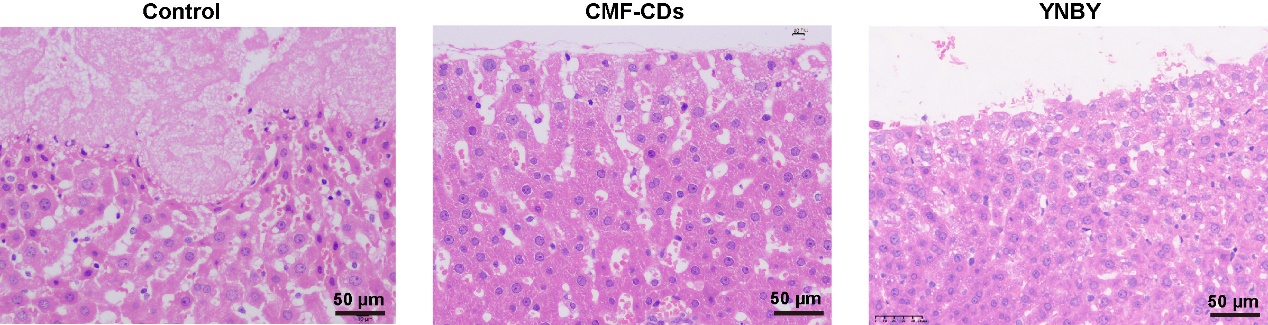
**

**Fig. S4.** H&E staining of liver wounds after CMF-CD treatment.


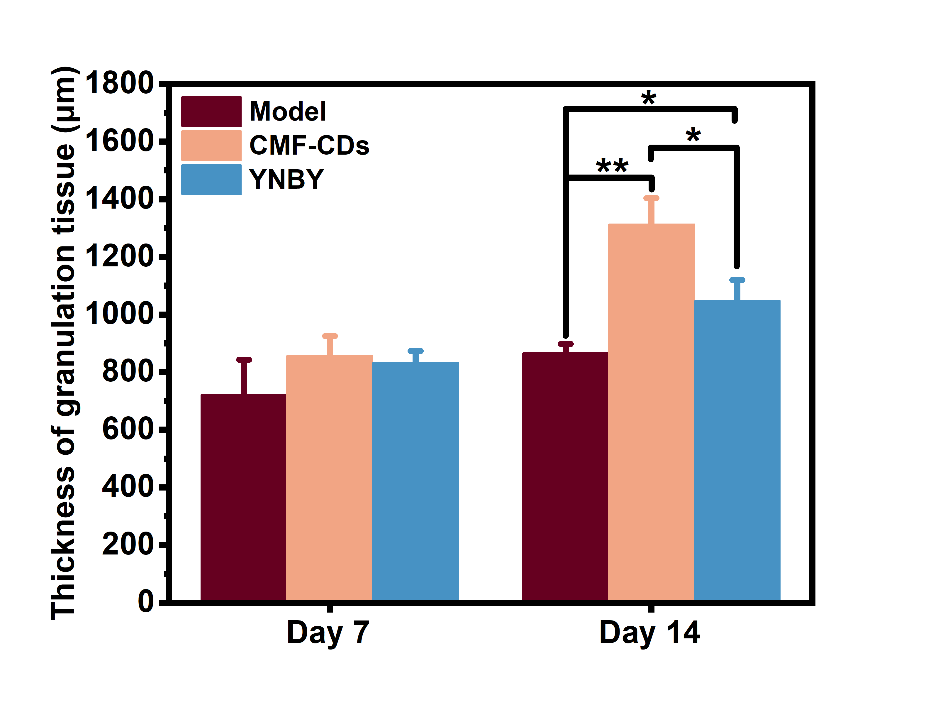


**Fig. S5.** Quantitative analysis of the thickness of granulation tissue after CMF-CD treatment (n = 3).


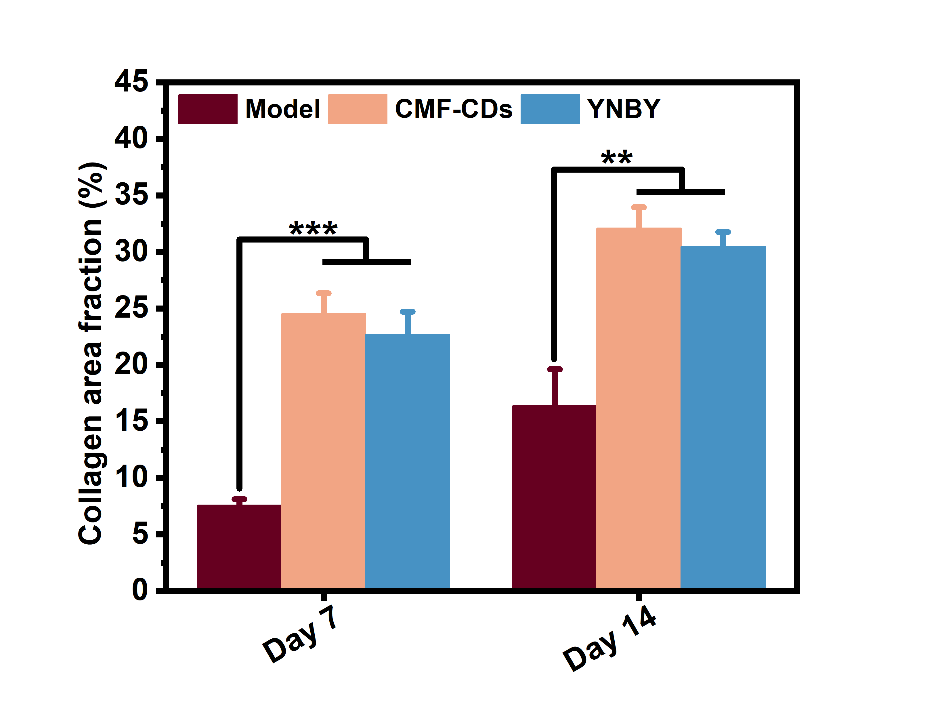


**Fig. S6.** Quantitative analysis of collagen area fraction (%) after CMF-CD treatment (n = 3).

**
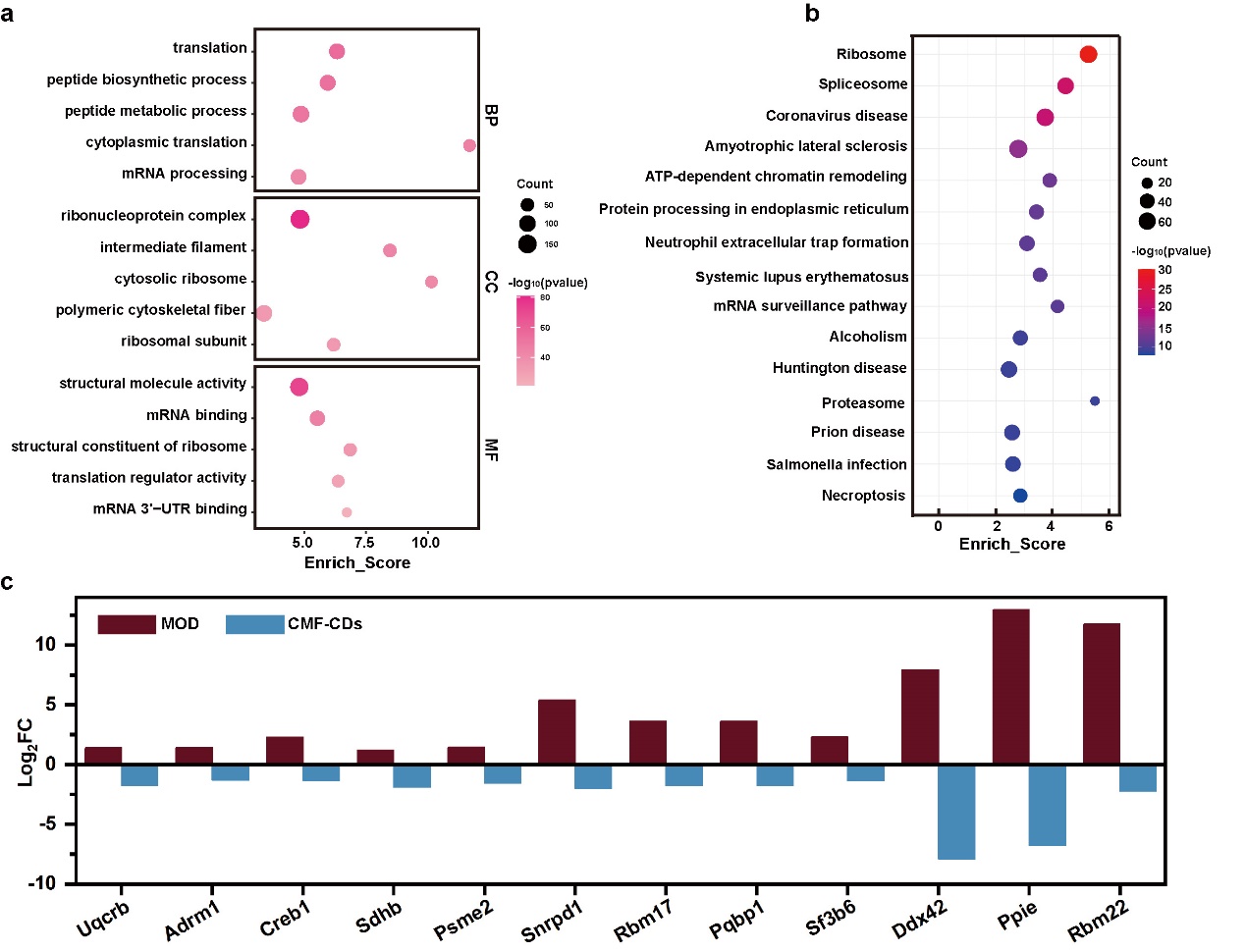
**

**Fig. S7.** a) GO and b) KEGG pathway enrichment analysis of the DEPs between the MOD and CON groups. c) The significantly downregulated proteins closely related to oxidative phosphorylation and tricarboxylic acid cycle metabolic pathways after CMF-CDs treatment.

**
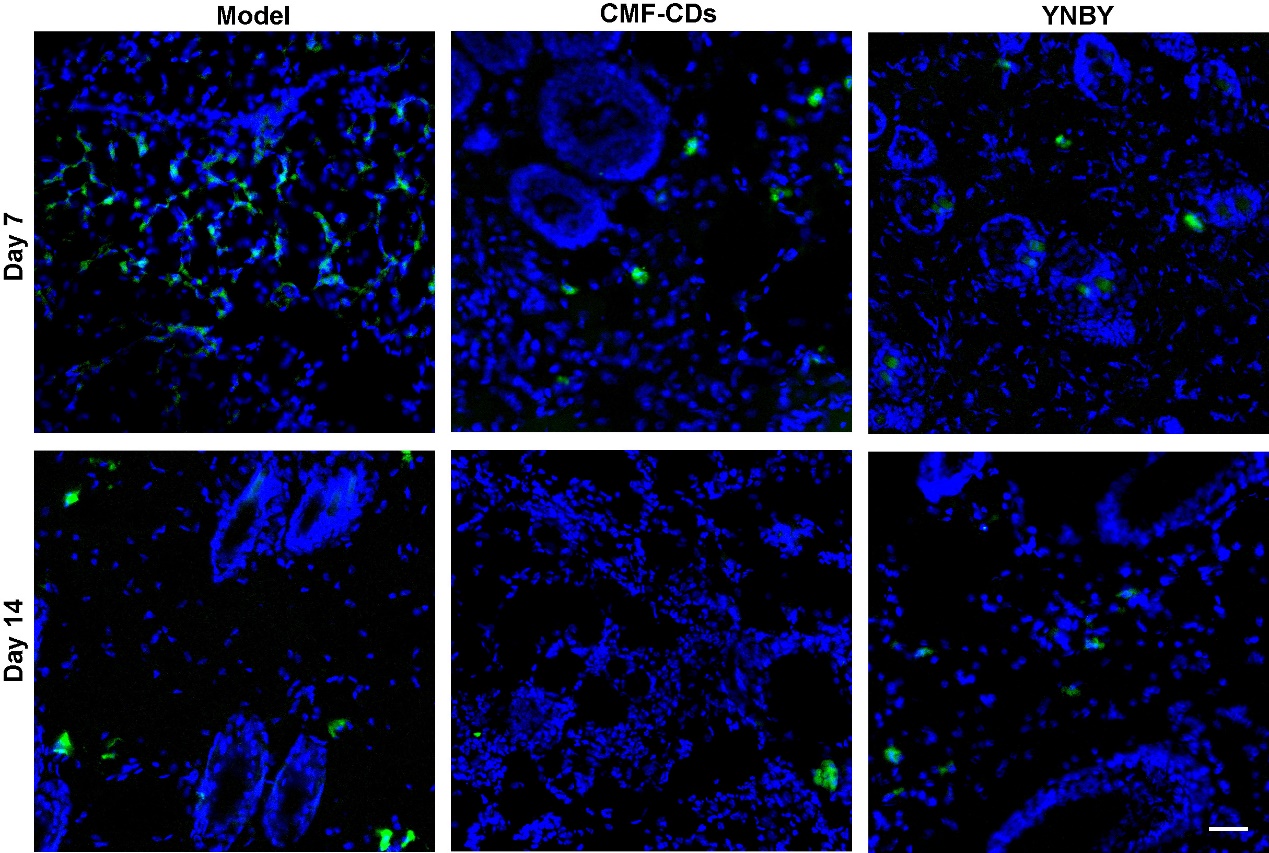
**

**Fig. S8.** Immunofluorescence staining of IL-6 expression in wound tissues on days 7 and 14 (scale bar, 100 μm).


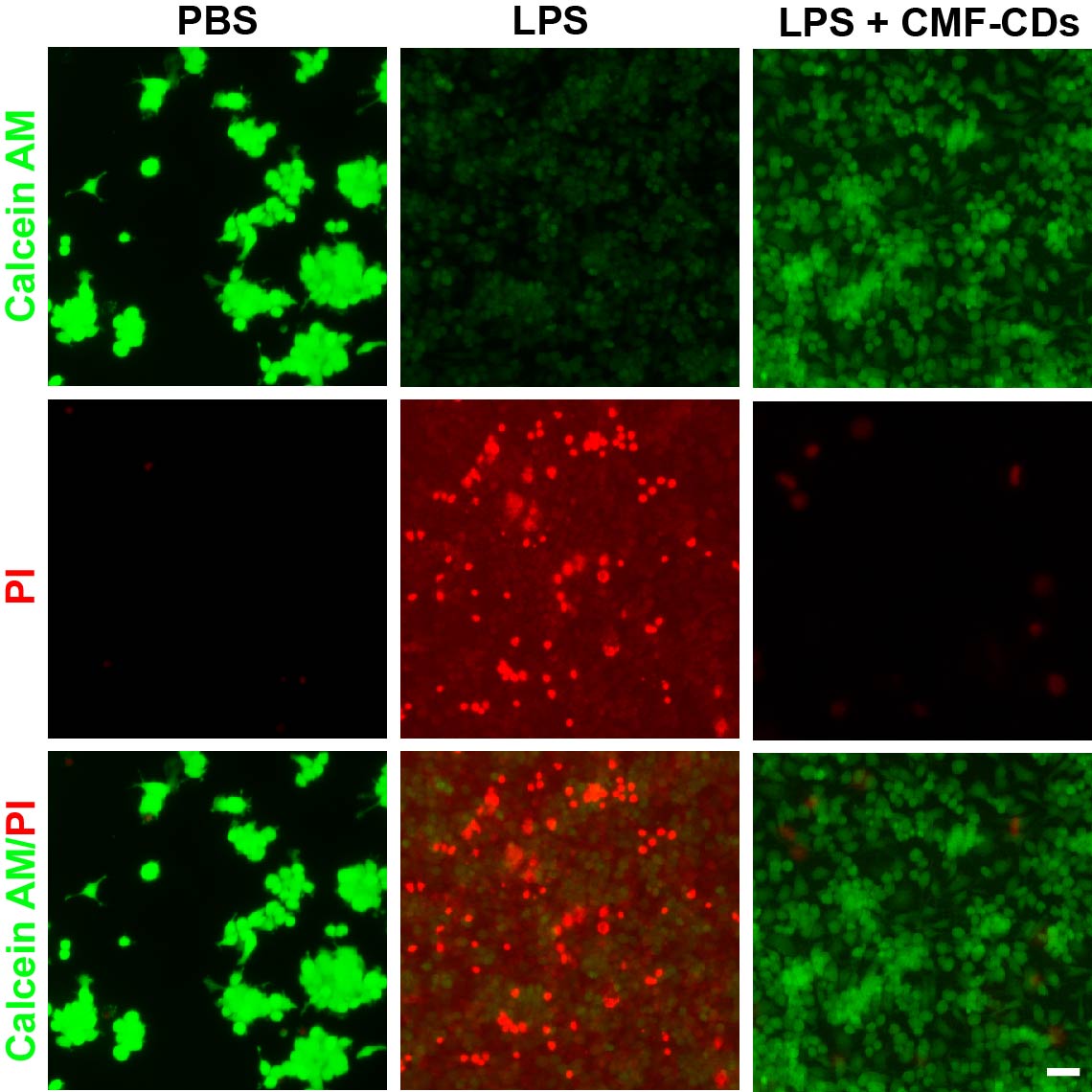


**Fig. S9.** Live/dead cell staining of RAW264.7 cells under oxidative stress (scale bar, 100 μm).

**References**

[1] T. Liu, B. Xiao, F. Xiang, J. Tan, Z. Chen, X. Zhang, C. Wu, Z. Mao, G. Luo, X. Chen, J. Deng, Ultrasmall copper-based nanoparticles for reactive oxygen species scavenging and alleviation of inflammation related diseases, Nat Commun 11(1) (2020) 2788.

[2] Q. Liu, Y. Ren, H. Jia, H. Yuan, Y. Tong, S. Kotha, X. Mao, Y. Huang, C. Chen, Z. Zheng, L. Wang, W. He, Vanadium Carbide Nanosheets with Broad-Spectrum Antioxidant Activity for Pulmonary Fibrosis Therapy, ACS Nano 17(22) (2023) 22527-22538.

[3] J. Liu, Z. Chen, H. Liu, S. Qin, M. Li, L. Shi, C. Zhou, T. Liao, C. Li, Q. Lv, M. Liu, M. Zou, Y. Deng, Z. Wang, L. Wang, Nickel-Based Metal-Organic Frameworks Promote Diabetic Wound Healing via Scavenging Reactive Oxygen Species and Enhancing Angiogenesis, Small (2023) e2305076.

[4] L. Ma, J.J. Zheng, N. Zhou, R. Zhang, L. Fang, Y. Yang, X. Gao, C. Chen, X. Yan, K. Fan, A natural biogenic nanozyme for scavenging superoxide radicals, Nat Commun 15(1) (2024) 233.

[5] L. Chen, M. Peng, J. Zhou, X. Hu, Y. Piao, H. Li, R. Hu, Y. Li, L. Shi, Y. Liu, Supramolecular Photothermal Cascade Nano-Reactor Enables Photothermal Effect, Cascade Reaction, and In Situ Hydrogelation for Biofilm-Associated Tooth-Extraction Wound Healing, Adv Mater 35(31) (2023) e2301664.
